# SpaJoint: a transfer learning method for spatial transcriptomics deconvolution

**DOI:** 10.1093/bib/bbag158

**Published:** 2026-04-08

**Authors:** Zichang Li, Xiangjie Li, Xiaokang Yu, Sheng Fang, Jingxiao Zhang, Xinyi Xu

**Affiliations:** Center for Applied Statistics, School of Statistics, Renmin University of China, 59 Zhongguancun Street, Haidian District, Beijing 100872, China; National Clinical Research Center for Cardiovascular Diseases, State Key Laboratory of Cardiovascular Disease, Fuwai Hospital, National Center for Cardiovascular Diseases, Chinese Academy of Medical Sciences and Peking Union Medical College, 167A Beilishi Road, Xi Cheng District, Beijing 100037, China; Department of Biostatistics, Epidemiology and Informatics, University of Pennsylvania, Blockley Hall, 423 Guardian Drive, Philadelphia, PA 19104, USA; Center for Applied Statistics, School of Statistics, Renmin University of China, 59 Zhongguancun Street, Haidian District, Beijing 100872, China; Center for Applied Statistics, School of Statistics, Renmin University of China, 59 Zhongguancun Street, Haidian District, Beijing 100872, China; School of Statistics and Mathematics, Central University of Finance and Economics, 39 South College Road, Haidian District, Beijing 100081, China

**Keywords:** spatial transcriptomics deconvolution, single-cell RNA sequencing, transfer learning, neighborhood graph

## Abstract

Currently, many widely used spatial transcriptomics (ST) technologies do not achieve single-cell resolution, with each spot capturing signals from multiple, potentially heterogeneous cells. As a result, a key challenge is to resolve the spatial distribution of distinct cell types within tissues, which is fundamental for understanding tissue architecture and biological function. Here we present a deconvolution method based on transfer learning, SpaJoint. This method integrates gene expression derived from single-cell RNA sequencing (scRNA-seq) and ST, taking into account the spatial correlation across locations of different spots. Comprehensive experiments demonstrate that SpaJoint achieves excellent performance in predicting the cell-type composition of spatial spots and identifying the spatial regions of cell types, thus highly effective and broadly applicable among various scRNA-seq and ST datasets. Additionally, it exhibits remarkable robustness to hyperparameters and provides significant advantage in computational efficiency.

## Introduction

The spatial position of cells within tissues and organs is crucial for their specific functions . In recent years, researchers have developed various spatial transcriptomics (ST) technologies that can capture transcriptional information from specific locations within a tissue. These advancements facilitate the study of cell subpopulations and the molecular mechanisms that play critical roles in tissue development and disease progression. However, there are two disadvantages in existing ST technologies: sequencing-based ST technology cannot achieve true single-cell resolution, e.g. 10x Visium [1, 2] and Slide-seq [3]; besides, imaging-based ST technology can only detect a limited amount of gene expression, e.g. seqFISH+ [4], MERFISH [5], STARmap [6], and Xenium [7]. To address the technological limitations, extensive literature has reported numerous algorithms that integrate ST data with single-cell RNA sequencing (scRNA-seq) data. These algorithms predict the spatial distribution of cell types and the full transcriptome information of individual cells. This integration provides deeper insights into ST data and their underlying biological processes.

Algorithms developed for the integrative analysis of ST and scRNA-seq data can be broadly classified into two core computational paradigms: (1) probabilistic model-based approaches formulate the deconvolution problem through statistical frameworks. The classic Seurat [8] method calculates the importance score indicating the probability of each cell type being present in each spot. Other methods include regression-style implementations like SpatialDWLS [9], SPOTlight [10], CARD [11], and Redeconve [12], as well as models based on probabilistic distributions such as NovospaRc [13], RCTD [14], SpatialScope [15], cell2location [16], DestVI [17], Stereoscope [18], STRIDE [19], and DOT [20]. (2) Deep learning-based approaches like Tangram [21], Spoint [22], DSTG [23], and CellDART [24] perform ST spot deconvolution by transferring cell-type information from scRNA-seq profiles. In addition, there are some methods that comprehensively utilize the above two paradigms, such as Celltrek [25] and CytoSPACE [26]. Besides deconvolution methods, SpaIM [27] leverages scRNA-seq data to predict unmeasured gene expressions in ST profiles. These studies demonstrate the significant potential of cross-modality integration, which leverage data information from one modality to support the analysis of another. Besides, scJoint [28] successfully transfers cell-type labels from scRNA-seq to scATAC-seq data through joint embedding and transfer learning, which greatly inspired the construction of the neural network in this paper. There are also reference-free deconvolution methods, such as STdeconvolve [29], performing ST deconvolution without scRNA-seq support. However, all these methods do not fully leverage the rich spatial localization information available in ST data, nor do they adequately account for the spatial structural heterogeneity of cell types. GraphST [30], as a method for spatial domain identification, utilizes the structural information in the ST part. However, when it serves as a deconvolution method, the model lacks supervised training for the scRNA-seq part. Therefore, it is necessary to develop a deconvolution algorithm that can effectively integrate the spatial location and structure information from ST data with the gene expression and label information from scRNA-seq data, thereby fully uncovering the biological information hidden in ST data.

In this study, we develop an algorithm for **joint**ly integrating **spa**tial and single-cell transcriptomic profiles based on the idea of transfer learning, named as SpaJoint, to estimate the cell-type composition of ST spots. A neural network is constructed to project ST and scRNA-seq data into a shared latent space, align them through similarity comparison, and finally analyze the cell-type proportion of spatial spots. Specifically, we build a neighborhood graph to encode local spatial structure, and introduce a location loss into the network to exploit both positional and structural information in the ST data. Compared with the state-of-the-art algorithms, SpaJoint performs better on a variety of commonly used simulated and real ST data. In addition, the computational time of SpaJoint is shorter than most deconvolution methods.

## Materials and methods

**Figure 1.**
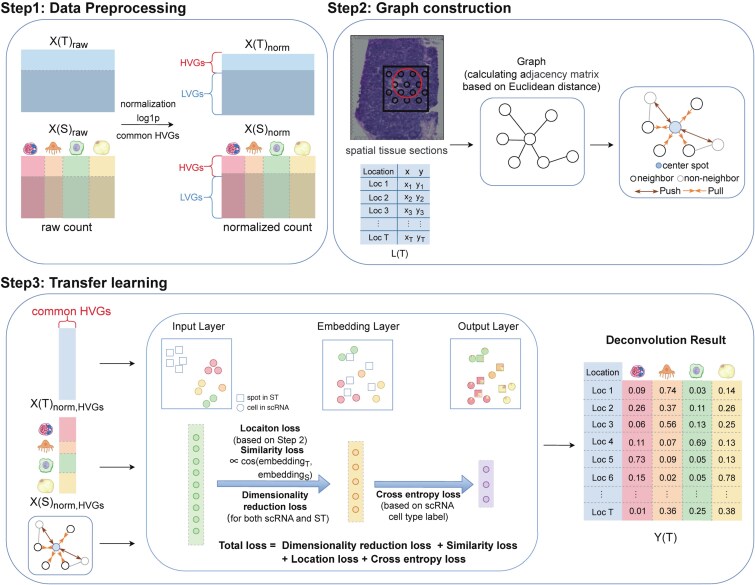
Overview of SpaJoint workflow for ST deconvolution.

Our method SpaJoint consists of data preprocessing, graph construction based on the spatial location of spots in ST data, and transfer learning based on neural network to achieve cell type deconvolution of spots. It adapts scJoint [28] for ST deconvolution by incorporating spatial architecture modeling inspired by GraphST [30]. The workflow of SpaJoint is illustrated in Fig. 1.

### Data preprocessing

Our method SpaJoint takes two gene expression matrices as input: a scRNA-seq matrix with cell-type annotations and an ST matrix containing spot location information. The scRNA-seq matrix is of dimension $N_{gene}^{S} \times N_{cell}$, and the ST matrix is of dimension $N_{gene}^{T} \times N_{spot}$, with $N_{gene}^{S} $, $N_{gene}^{T}$, $N_{cell}$, and $N_{spot}$ denoting the number of scRNA-seq genes, ST genes, cells, and spots, respectively. First, we apply normalization and log-transform to the scRNA-seq and ST matrices separately. Then, we identify highly variable genes (HVGs) separately in scRNA-seq data and ST data, and take the intersection of the two sets as common HVGs. After data preprocessing, the scRNA-seq matrix of dimension $N_{hvg} \times N_{cell}$ and the ST matrix of dimension $N_{hvg} \times N_{spot}$ are fed into neural network, with $ N_{hvg}$ denoting the number of common HVGs.

### Graph construction for ST data

ST data offer a key advantage in its ability to capture spatial context, enabling identification of cells with correlated expression profiles and spatial locations. To leverage this spatial information, we constructed an undirected neighborhood graph $G=(V,E)$, where $V$ denotes the set of spots and $E$ represents the edges linking spatially adjacent spots. In this graph, each node is connected to its $m$ spatially nearest neighbors. Thus, the spatial neighborhood of each spot is derived by its coordinate-based distances to other spots. The topological structure of $G$ is encoded in an adjacency matrix $A \in R^{N_{spot} \times N_{spot}}$, where $a_{ij}=1$ indicates that spot $j$ lies within the neighborhood of spot $i$, and $a_{ij}=0$ otherwise. To better adapt to the characteristics of ST data, we use the *gr.spatial_neighbors()* function from the python package Squidpy to search for the neighbors of each spot. For non-Visium data, we set the parameters *coord_type=“generic”* and *n_neighs=m*, where *n_neighs* specifies the fixed number of neighbors for each spot. For Visium data, we set the parameters *coord_type=“grid”* and *n_rings*, which specifies how many hexagonal rings around each spot will be considered as neighbors.

### Transfer learning based on neural network

Our method SpaJoint utilizes a well-annotated scRNA-seq data as reference to estimate the abundance of various cell types in each spot of ST data. We consider an scRNA-seq dataset of $S$ cells, comprising preprocessed expression profiles $\{\mathbf{x}_{s}\}_{s=1}^{S}$ and cell-type labels $\{y_{s}\}_{s=1}^{S}$, where $y_{s} \in \{1, \ldots , K\}$ and $K$ is the number of cell types. Similarly, the ST data consist of $T$ unlabeled spots with preprocessed expression vectors $\{\mathbf{x}_{t}\}_{t=1}^{T}$. The length of vector $\mathbf{x}_{s}$ and $\mathbf{x}_{t}$ is equal to the number of common HVGs.

The neural network in SpaJoint consists of one input layer, one embedding layer, and one output layer. Its trainable weights and biases are denoted collectively by a parameter set $\theta $. With the two modalities now aligned on the number of features in the input layer, we co-train them using a shared encoder, thereby enabling weight sharing. The embedding layer contains $D$ neurons with linear activation, which capture aligned features between cells and spots, thus acting as the joint low-dimensional embedding space. In this layer, the transformation $f$ maps input $\mathbf{x}_{s}$ to a embedding vector $\mathbf{f}_{\theta ,s}=f(\mathbf{x}_{s};\theta ) \in \mathbf{R}^{D}$. The output layer has dimension matching the number of cell types in the scRNA-seq data. By applying a softmax transformation, this layer generates a vector $\mathbf{g}_{\theta ,s}=softmax (h(f(\mathbf{x}_{s};\theta ))) \in \mathbf{R}^{K}$ representing the predicted cell-type proportions at each cell, where $h$ means the mapping function of the output layer. The same transformation is applied to ST data $\mathbf{x}_{t}$ to obtain corresponding embedding $\mathbf{f}_{\theta ,t}$ and probability vector $\mathbf{g}_{\theta ,t}$.

We employ the minibatch training approach to enhance computational efficiency and optimize the convergence performance of model training. A minibatch $\mathbf{\mathcal{B}}$ is created by sampling equal-sized subsets of cells (or spots) from each modality, i.e. $\mathbf{\mathcal{B}}^{(s)}$ with $B$ cells from scRNA-seq data and $\mathbf{\mathcal{B}}^{(t)}$ with $B$ spots from ST data. The training process is divided into two phases:

**Phase 1: from the input layer to the embedding layer**, we simultaneously achieve dimension reduction and alignment of scRNA-seq and ST embeddings by applying appropriate loss functions. The loss function at this phase is composed of losses (1)–(3).

**Dimensionality reduction loss** (for scRNA-seq and ST separately). To obtain the low-dimensional embeddings, we employ a dimensionality reduction loss function that shares a similar underlying principle with principal component analysis (PCA). Given the input vector $\mathbf{x}_{b}$, define $\mathbf{f}_{\theta ,b}=f(\mathbf{x}_{b},\theta ) \in R^{D}$, and $\overline{\mathbf{f}}_{\theta ,\cdot } = \frac{1}{B}\sum _{b \in \mathbf{\mathcal{B}}} \mathbf{f}_{\theta ,b} \in R^{D}$, where $b$ denotes $s$ for scRNA-seq data and $t$ for ST data. We denote $f_{\theta }(j)$ as the $j$th coordinate of vector $\mathbf{f}_{\theta }$, and $\sum _{\theta ,.}$ as the sample correlation matrix among the coordinates of vector $\mathbf{f}_{\theta ,b}$. The loss is defined as 


(1) \begin{align*} \mathcal{L}_{reduction}(\theta)&=\left(\frac{1}{BD}\sum_{b \in \mathbf{\mathcal{B}}}\sum_{j=1}^{D}|f_{\theta,b}(j)-\overline{f}_{\theta,\cdot}(j)|\right)^{-1}\notag\\&+\frac{1}{D^{2}}\sum_{i \neq j}|\Sigma_{\theta,\cdot}(i,j)|+\frac{1}{D}\sum_{j=1}^{D}|\overline{f}_{\theta,\cdot}(j)|.\end{align*}

In the optimization of this loss, the first term aims to maximize the variation of each coordinate, and the second term aims to achieve orthogonality by minimizing the correlation between coordinates. By penalizing the $L1$ norm of the embeddings, the third term serves as a regularization term and constrains the mean of each coordinate to be near zero. In this way, the dimensionality reduction loss achieves the classical objectives of PCA while preserving the flexibility of neural networks.

**Cosine similarity loss**. This loss aims to maximize the similarity between aligned ST and scRNA-seq embedding vector pairs. First, for each $t \in \mathbf{\mathcal{B}}^{(t)}$, we find the corresponding $s \in \mathbf{\mathcal{B}}^{(s)}$ that maximizes $cos(\mathbf{f}_{\theta ,t},\mathbf{f}_{\theta ,s})$. Then we take an average of the maximum similarity over all $t$. The loss is given by 


(2) \begin{align*}& \mathcal{L}_{cos}(\theta)=-\frac{1}{B}\sum_{t \in \mathbf{\mathcal{B}}^{(t)}} \mathop{max}\limits_{s \in \mathbf{\mathcal{B}}^{(s)}}cos(\mathbf{f}_{\theta,t},\mathbf{f}_{\theta,s}),\end{align*}

where function $cos(\cdot )$ is the cosine similarity defined as $cos(\mathbf{u},\mathbf{v})=\langle \mathbf{u},\mathbf{v} \rangle /(\Vert \mathbf{u} \Vert \Vert \mathbf{v} \Vert )$ for a pair of vectors $(\mathbf{u}, \mathbf{v})$. This loss serves a dual purpose: first, to globally align the two modalities and bring them closer together in the embedding space; second, a basis for subsequent label transfer, to ensure that each spot receives at least one cell type label from its best-matching cell in the scRNA-seq reference.

**Location loss**. It is widely accepted that cellular states are influenced by their local microenvironment, leading to the observation that adjacent cells often have similar expression profiles, no matter in the original data space or in the dimension-reduced embedding layer. To integrate the information of sequencing and coordinates in ST data, as described in Section 2.2, we convert the spatial coordinates into an undirected graph $G=(V,E)$ and define the adjacency matrix $A \in R^{T \times T}$ accordingly. We then combine the spatial structure information with the embedding layer output $\mathbf{f}_{\theta ,t}$ from ST data, and define the location loss as 


(3) \begin{align*}& \mathcal{L}_{loc}(\theta)=-\frac{1}{B}\sum_{i=1}^{B}\sum_{j \in \mathcal{N}_{i}}log\frac{exp(cos(\mathbf{f}_{\theta,i},\mathbf{f}_{\theta,j}))}{\sum_{p \notin \mathcal{N}_{i}}exp(cos(\mathbf{f}_{\theta,i},\mathbf{f}_{\theta,p})))},\end{align*}

where $\mathcal{N}_{i}$ denotes the set of spatial neighbors of spot $i$. This loss is driven from the concept of contrastive learning, which is designed to maximize similarity between positive pairs while minimizing similarity between negative pairs. For each spot $i$, we split other spots as positive or negative samples based on whether they fall within its neighborhood, where positive pairs are drawn from its adjacent spots, and negative pairs are constructed with nonadjacent spots.

All the above loss functions, $\mathcal{L}_{reduction}$, $\mathcal{L}_{cos}$, and $\mathcal{L}_{loc}$, are used to calculate parameters from the input layer to the embedding layer, jointly yielding a low-dimensional representation. This combined loss is termed the representation loss and is formulated as 


(4) \begin{align*} \mathcal{L}_{representation}(\theta) =& w_{1}\mathcal{L}_{reduction}^{scRNA}(\theta) + w_{2}\mathcal{L}_{reduction}^{ST}(\theta)\notag \\&+ w_{3}\mathcal{L}_{cos}(\theta) + w_{4}\mathcal{L}_{loc}(\theta),\end{align*}

where the reduction loss is calculated separately for scRNA-seq and ST gene matrix. $w_{1}$–$w_{4}$ denote the user-specified weights for the respective loss terms and sum to $1$. Typically, we consider dimensionality reduction for the scRNA-seq and ST data to be equally important, i.e. $w_{1} = w_{2}$.

**Phase 2: from the embedding layer to the output layer**, we perform supervised training using scRNA-seq data with cell-type labels. The trained network then takes ST embedding $\mathbf{f}_{\theta ,t}$ to predict $\mathbf{g}_{\theta ,t}$, which implies the cell-type composition for each spot. In this way, the scRNA-seq labels have been successfully transferred to ST data, which completes transfer learning. This phase utilizes only a cross entropy loss.

**Cross entropy loss**. This loss is computed by comparing the predicted cell-type proportions from scRNA-seq data with the ground-truth annotations, as defined below: 


(5) \begin{align*}& \mathcal{L}_{entropy}(\theta) =-\frac{1}{B}\sum_{s \in \mathbf{\mathcal{B}}^{(s)}}\sum_{k=1}^{K}\mathbf{1}(y_{s}=k)log(g_{\theta,s}(k)),\end{align*}

where $y_{s}$ is the true cell-type annotation, $\mathbf{1}(\cdot )$ is an indicator function, and $g_{\theta ,s}(k)$ is the $k$th element of probability vector $\mathbf{g}_{\theta ,s}$.

Here, $\mathcal{L}_{entropy}$ is used to calculate parameters from the embedding layer to the output layer. Accordingly, to obtain the optimal estimate of the parameter set $\theta $, we minimize the following final loss function over each minibatch, which can be expressed as 


(6) \begin{align*}& \mathcal{L}(\theta) = \mathcal{L}_{representation}(\theta) + \mathcal{L}_{entropy}(\theta).\end{align*}

After the set of parameters $\theta $ of the neural network is determined by the above loss functions, the result of the output layer $\mathbf{g}_{\theta ,t}$ is the cell abundance, i.e. a vector of predicted proportions for all cell types, in each spot.

### Unique contributions

The unique contribution of SpaJoint lies in effectively integrating spatial structural information from ST data and cell type information from scRNA-seq data. Inspired by the advantage of scJoint in integrating two modalities, SpaJoint enhances the neural network by incorporating a critical location loss, to adapt it for the ST deconvolution task. The location loss, which consists of constructing a spatial neighborhood graph and performing contrastive learning between adjacent and nonadjacent spots, is widely used for different purposes. Uniquely, we employ it here to ensure that spatially close spots have similar representations in the embedding layer, thereby optimally preserving the local spatial structure of ST data in the neural network. With this framework, SpaJoint achieves superior performance in benchmark comparison.

### Performance evaluation

#### Experimental setup

We selected top 2000 HVGs and used their expression profiles as features for each cell and spot. In the graph construction, we selected $m$ nearest neighbors for each spot. SpaJoint achieved its best performance in most of non-Visium datasets with $m$ set to 6. For Visium datasets, to ensure computational efficiency, we suggested not considering hexagonal rings beyond 2, and *n_rings=2* often performed better. In the neural network, we set the dimension of the embedding layer $D$ as 64, the batch size as 256 (128 for ST data with a small number of spots) and the epoch as 100. For the loss functions, the optimal combination of weights was determined through grid search, with $w_{1}$, $w_{2}$, $w_{3}$, and $w_{4}$ being 0.3, 0.3, 0.2, and 0.2, respectively. A sensitivity analysis of the hyperparameters is discussed in detail in Section 3.4.

#### Evaluation metrics

To show the effectiveness of SpaJoint for ST cell-type deconvolution with scRNA-seq data, we compared SpaJoint with 11 state-of-the-art methods, including RCTD, Spoint, Tangram, DOT, cell2location, CellDART, GraphST, CARD, DestVI, SpatialScope, and STdeconvolve. Notably, SpatialScope is a method for cell-type annotation of spots rather than deconvolution, while STdeconvolve is a reference-free method that can only be compared on simulated data. We transformed the outputs of these two methods so that they can be used for benchmarking, with details elaborated in Supplementary Section S1.2. To comprehensively compare the deconvolution performance of different methods, four metrics were used: Pearson correlation coefficient (PCC), structural similarity (SSIM), root mean square error (RMSE), and Jensen–Shannon divergence (JSD). PCC calculates the correlation between the true and estimated values. SSIM measures the structural similarity when comparing two matrices. RMSE quantifies the dispersion between the true and predicted values. JSD uses Kullback–Leibler divergence to measure the difference between two distributions. Therefore, all these metrics can be employed to evaluate the prediction accuracy of the deconvolution results. We calculated these four metrics separately for spots and cell types, where higher PCC and SSIM as well as lower RMSE and JSD indicated better deconvolution performance. We also integrated the four metrics into a new measure—the Mean Rank—to comprehensively evaluate performance across these metrics. The method with the lowest Mean Rank, i.e. ranked first, demonstrates the best performance among all integration approaches. The detailed calculation of all metrics is described in Supplementary Section S1.1.

#### Datasets

To validate the performance of SpaJoint, we applied it to seven datasets including three simulated data and four real data. The simulation study employs three datasets with synthetic ground truth to quantitatively benchmark SpaJoint against competing methods. Mouse visual cortex (STARmap) data and mouse cortex (seqFISH+) data are obtained from the same benchmark study [31], sharing a common scRNA-seq reference but profiled with different ST technologies. Mouse brain (Stereo-seq) data are a **large-scale** dataset used to assess SpaJoint’s scalability. With respect to real datasets, human lymph node (10x Visium) data and mouse hippocampus (Slide-seqV2) data were used to compare the effectiveness of all methods on empirical data. On human breast cancer (10x Visium) data and chicken heart development (10x Visium) data, the deconvolution results have biological interpretations supported by prior studies, enabling validation of SpaJoint’s biological plausibility. The detailed data description is provided in Supplementary Section S2.

## Results

### Benchmarking on simulated data

**Figure 2.**
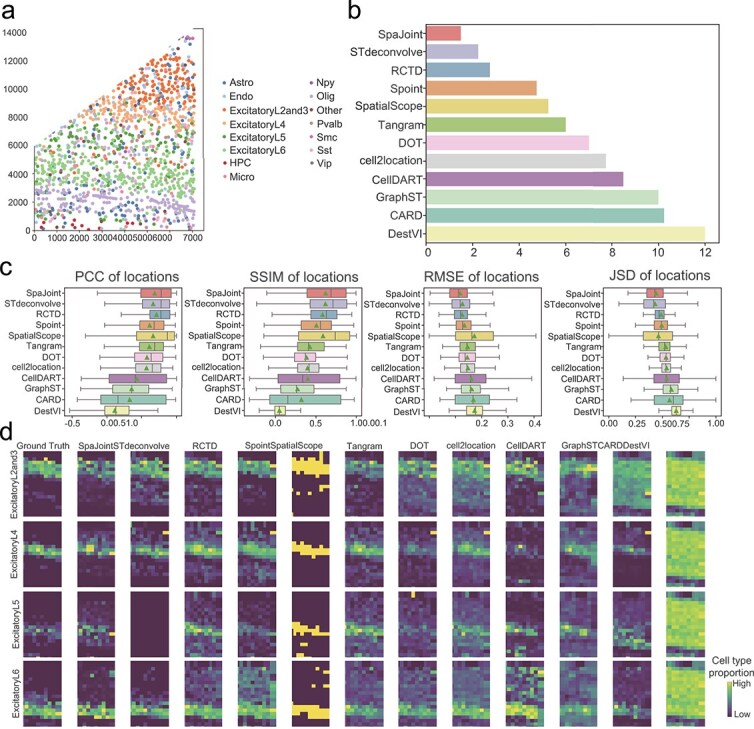
Performance benchmarking with mouse visual cortex STARmap dataset. **a**. A STARmap slide of mouse visual cortex, with spots annotated by cell types. **b**. Bar plots of Mean Rank (aggregated from PCC, SSIM, RMSE, and JSD) at the spot level. **c**. Boxplots of PCC, SSIM, RMSE, and JSD of each deconvolution method in predicting the cell-type proportion of spots. Center line: median; box limits: upper and lower quartiles; whisker: 1.5 $\times $ interquartile range; triangle: mean value; number of predicted spots: 189. Higher PCC and SSIM, lower RMSE and JSD indicate better performance. **d**. The proportion of major cell types in the spots, including the ground truth and the predicted results of 12 methods.

**Mouse visual cortex STARmap data.** In this dataset, the cell type label of each spot is reported in Fig. 2a. We generated pseudo-spots from the original data (details in Supplementary Section S2) and performed deconvolution. We evaluated the prediction accuracy at both the spot and cell-type levels using metrics PCC, SSIM, RMSE, and JSD. Across all metrics, our method shows a clear advantage over other methods (Fig. 2c, Supplementary Fig. 1a–c). Based on the average ranking of all metrics, SpaJoint performs the best over all competing methods at the spot level (Fig. 2b). For layers or cell types with distinct spatial structures, the deconvolution plots in Fig. 2d display the predicted cell-type proportions at each spot. SpaJoint achieves the best alignment with the ground truth, confirming its superior accuracy in predicting cell-type distributions. Consistently superior performance of SpaJoint is observed in other cell types as well (Supplementary Fig. 1d).

**Mouse cortex seqFISH+ data.** The deconvolution results of these data are shown in Supplementary Fig. 2. At the spot level, SpaJoint, CARD, and cell2location rank among the top three, while at the cell-type level, CARD, RCTD, and SpaJoint take the top three positions. Whether at the spot level or the cell-type level, SpaJoint stably remains a top-performing method across all metrics.

**Mouse brain Stereo-seq data.** Benchmark results of these data in Supplementary Fig. 3 reveal that cell2location and SpaJoint achieve superior performance, while Tangram, RCTD, and Spoint show intermediate accuracy, and the rest methods yield poor deconvolution outcomes. Excellent performance on this large-scale dataset further validates the scalability of SpaJoint.

Based on the results from these three simulated datasets, SpaJoint consistently ranks among the top-performing methods across all benchmark comparison and demonstrates robust performance with different sequencing technologies.

### Performance comparison on real data

**Figure 3.**
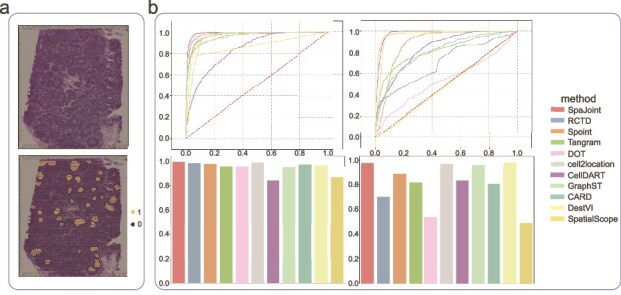
Performance on the human lymph node dataset. **a**. Top: H&E image of human lymph node data. Bottom: GC area from the cell2location study. **b**. Top: ROC curves. Bottom: AUC value of two cell types (B_Cycling, and B_GC_DZ) localized in the GCs.

**Human lymph node data.** The ST component of this dataset was acquired with germinal centers (GC). The ground-truth annotations for the GC were sourced from the original cell2location study [16] (Fig. 3a). We conducted a visual comparison by examining how each method mapped cell types to GC and non-GC locations. Using whether a spot is actually in the GC as the true label, if the predicted proportion of one subtype of B cells exceeds a certain threshold such as 0.5 in this spot, it is classified as GC; otherwise, it is classified as non-GC. Based on the true labels and predicted labels, we calculated the true positive rate and false positive rate to plot the receiver operating characteristic (ROC) curve, and the area under the curve (AUC) was determined accordingly. Higher AUC value indicates greater likelihood that the predicted B cells are truly located in the GC region. SpaJoint more accurately maps B cells, such as cycling B cells (B_Cycling) and dark zone B cells (B_GC_DZ), to the annotated GC locations, no matter from the deconvolution plots, the ROC curves, or the AUC value (Fig. 3b). Additionally, B_Cycling and B_GC_DZ are correctly colocalized on the GC region based on SpaJoint deconvolution, which has been also confirmed in the previous study [32]. Visualization of B-cell prediction proportions across spots (Supplementary Fig. 4) shows SpaJoint’s superior edge detection capability in spatial reconstruction.

**Figure 4.**
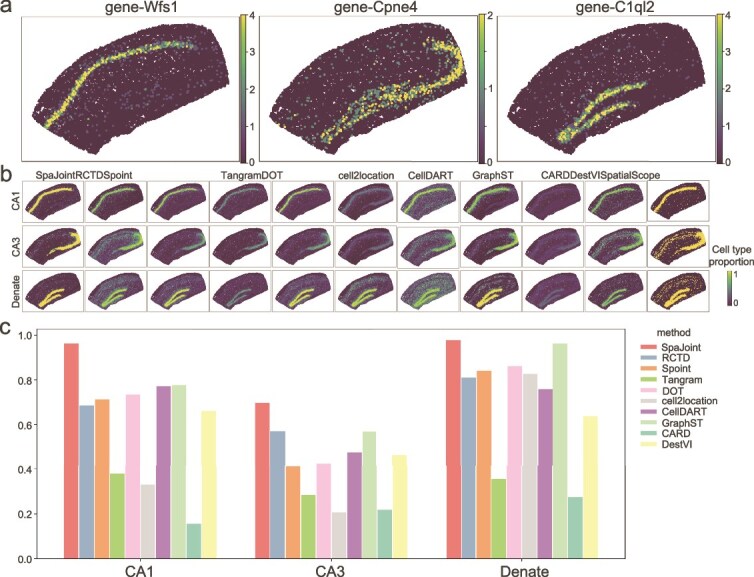
Analysis of the mouse hippocampus region. **a**. The expression levels of corresponding cell-type-specific marker genes. The examined cell types are CA1 cells, CA3 cells, and Dentate cells. **b**. The proportions of major cell types inferred by 11 methods are displayed on the spatial location. **c**. The mean cell-type proportion of CA1, CA3, and Dentate on the spots where the expression of the corresponding marker genes is greater than 1.

**Mouse hippocampus data.** For these data, the deconvolution result of SpaJoint perfectly matches the distribution of corresponding marker genes of three important cell types (CA1: Wfs1, CA3: Cpne4, Dentate: C1ql2) (Fig. 4a, b). We calculated mean proportions of CA1, CA3, and Dentate on the spots where the expression of corresponding marker genes is greater than a specified threshold, further verifying more accurate deconvolution by SpaJoint than the other methods (Fig. 4c, Supplementary Fig. 5). Comparative evaluation on two real datasets reveals SpaJoint’s superior deconvolution performance against nine competing methods. Since the output of SpatialScope is binary (i.e. a one-hot vector with 1 at the entry of annotated cell type and 0 elsewhere), we excluded it from the comparison in Fig. 4C and Supplementary Fig. 5.

### Biological validation on real data

**Figure 5.**
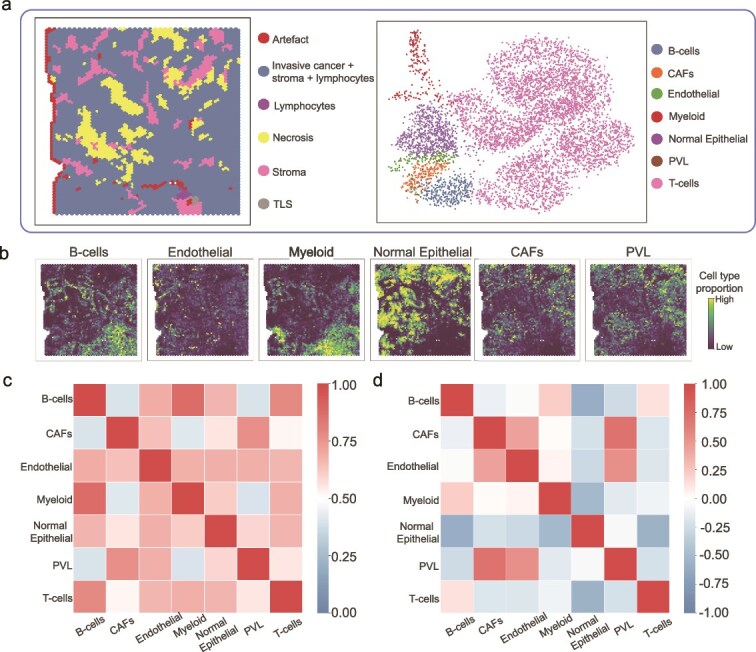
SpaJoint application to human breast cancer data. **a**. Left: the detected spatial domains of the ST data. Right: the UMAP of seven cell types in the scRNA-seq data. **b**. SpaJoint deconvolution result of six major cell types. The correlation matrix (using PCCs) **c**, of the average gene expression and **d**, of the predicted proportions across all spots between each pair of cell types.

**Human breast cancer data.** In this dataset, the ST data have six detected spatial domains and the corresponding scRNA-seq data contain seven distinct cell types (Fig. 5a). From Fig. 5b, the co-localization of B cells and Myeloid cells, as well as the co-localization of CAFs and PVL in the tumor microenvironment, can be clearly observed. Meanwhile, the spatial locations of Normal Epithelial cells are completely different from B cells and Myeloid cells. These phenomena have been confirmed in previous studies [33–35]. We also calculated the correlation matrix (using PCCs) of the average gene expression between each pair of cell types, as well as the correlation matrix of the predicted proportions across all spots between each pair of cell types (Fig. 5c, d). The consistency between these two matrices reflects the concordance between gene expression and spatial expression. Following spatial deconvolution, we performed cell–cell interaction analysis. The details about downstream analysis in Supplementary Section S4 further demonstrate that our deconvolution results are biologically meaningful.

**Figure 6.**
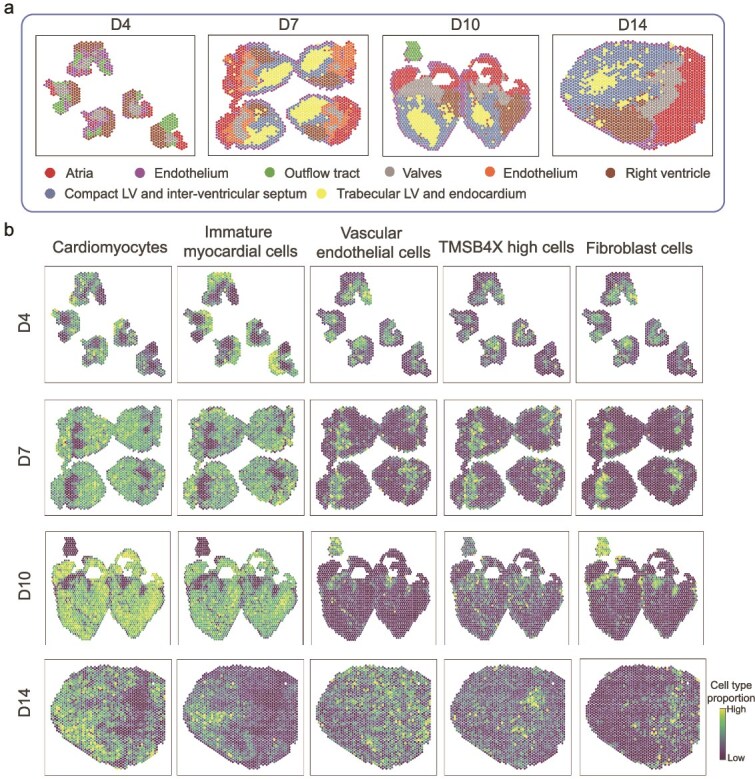
SpaJoint application on developmental chicken heart data. **a**. The chicken heart structure development during D4, D7, D10, and D14, colored by spatial regions of the ST data. **b**. SpaJoint deconvolution result: proportion of five major cell types (labels from scRNA-seq data) in D4, D7, D10, and D14.

**Chicken heart development data.** In these data, spatial domains are defined for each time point, and the aggregation of distinct cardiac chambers is readily discernible, as illustrated in Fig. 6a. SpaJoint demonstrates precise spatial mapping of five major cell types across cardiac developmental stages. This spatial analysis reveals two key histological distributions: first, cardiomyocytes show predominant localization in non-valvular myocardial regions [36]. Second, valvular structures are principally composed of fibroblast populations [37, 38] (Fig. 6b). In summary, SpaJoint precisely reconstructs cell-type spatial organization, providing critical insights into tissue biology.

### Sensitivity analysis and computational efficiency

**Figure 7.**
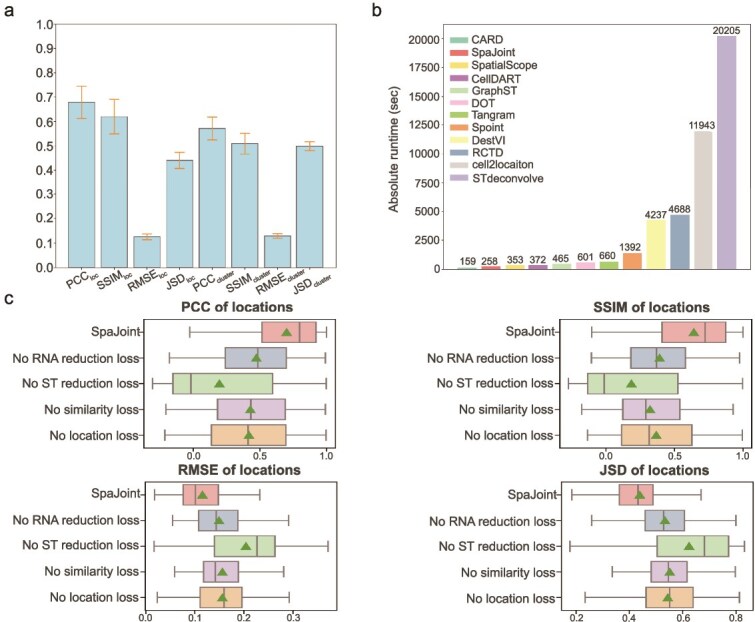
Sensitivity analysis and runtime. **a**. Bar plots of eight different metrics (PCC, SSIM, RMSE, JSD for locations and clusters separately) for mouse visual cortex STARmap data. For each hyperparameter combination, the mean of each metric over all locations or clusters is computed. The bar height is the average and the error bar is the standard deviation across the means of 80 combinations. **b**. Computational efficiency of different algorithms on the human lymph node dataset. **c**. The ablation study on mouse visual cortex STARmap dataset comparing the original SpaJoint and the other four conditions: no scRNA-seq reduction loss, no ST reduction loss, no similarity loss, and no location loss. Boxplots of PCC, SSIM, RMSE, and JSD of the above five conditions in predicting the cell-type distribution of spots. Higher PCC and SSIM, and lower RMSE and JSD indicate better performance.

We conducted comprehensive sensitivity analysis testing several key hyperparameters, including the number of HVGs (1000, 2000, 3000, 5000), the number of neighbors (4, 6, 8, 12), and the weights of loss functions (Supplementary Table 4), which yielded 4 $\times $ 4 $\times $ 5 = 80 distinct hyperparameter combinations. Performance on three simulation datasets indicates that SpaJoint remains remarkably stable across a wide range of parameter variations (Fig. 7a, Supplementary Fig. 6). Then we conducted ablation study to explore the contribution of different loss parts in SpaJoint on the previous mouse visual cortex dataset. We considered four condition: no scRNA-seq reduction loss, no ST reduction loss, no similarity loss, and no location loss. We utilized PCC, SSIM, RMSE, and JSD under various conditions relative to the original SpaJoint to indicate the significance of each loss function. It is evident that ST reduction loss dominates the prediction accuracy, followed by the location loss, which also shows substantial impact and serves as the main innovation of SpaJoint algorithm (Fig. 7c). We also conducted a series of additional sensitivity analyses, specifically targeting various parameters and uncertainty scenarios, on the mouse visual cortex STARmap dataset; the details are provided in Supplementary Section S3.2. Furthermore, we compared the absolute runtime of SpaJoint on human lymph node data with 11 other methods, on a Linux server with 32 CPU cores, 251 GB of RAM, and NVIDIA TITAN V GPUs. SpaJoint shows a leading advantage in computational speed in Fig. 7b. In addition, we have also included the maximum CPU and GPU memory used by each method in Supplementary Table 3. Our method supports GPU resources for practical use, and it demonstrates moderate utilization of both GPU and CPU resources.

## Discussion

In this article, we propose a deconvolution method based on transfer learning named SpaJoint. It leverages the spatial information from ST data to construct a neighborhood graph and an adjacency matrix, projects scRNA-seq and ST data into the same latent space, and utilizes the similarity of gene expression to analyze the cell types composition in the spots. In particular, we consider the principle that spots close in the spatial location should express similarly in the latent embedding space, effectively integrating spatial location information into the neural network.

Our comprehensive experiments show that SpaJoint is highly effective on various types of scRNA-seq and ST data, and also has significant advantage in computational efficiency. SpaJoint is highly flexible and can be applied to any type of single-cell and ST data, without any requirements on the data distribution. Unlike other methods requiring stringent quality control, SpaJoint maintains robust performance even with highly imbalanced datasets. For example, CellDART and cell2location require strict data preprocessing, which sometimes filters out certain spots and leads to incomplete deconvolution results. RCTD, when applied to human breast cancer data, failed to identify cell-type PVL, and in human lymph node data deconvolution, it missed cell-type Mast. In contrast, SpaJoint identifies all cell types present in the scRNA-seq data, and is therefore capable of preserving rare cell populations.

Despite the notable achievements of the proposed method, several limitations remain to be addressed. Primarily, adaptive tuning of key hyperparameters (such as the number of neighbors and loss weights) through strategies like cross validation is expected to improve the performance of our method on specific datasets. Meanwhile, more data characteristics (such as data sparsity, data scale, heterogeneous spatial structure) remain to be further addressed in the future. Additionally, even though an increasing number of approaches are leveraging scRNA-seq data to offer more detailed transcriptional insights for ST data, scRNA-seq and ST data are typically not derived from the same tissue section, introducing inherent inconsistency and potential batch effect that can impact the deconvolution outcomes. Moreover, when there is a significant difference between the number of cells in scRNA-seq data and the number of spots in ST data, it is necessary to set a higher number of epochs to better align the data. In the future, we will also consider combining deconvolution methods with gene imputation approaches such as SpaIM to enhance the integrated analysis of scRNA-seq and ST data.

**Key Points**

- SpaJoint achieves accurate spatial deconvolution by integrating spatial information from ST data with cell-type labels from scRNA-seq data.
- SpaJoint constructs a neighborhood graph to encode local spatial structure, and then introduces a location loss into the neural network to exploit both positional and structural information in the ST data.
- SpaJoint demonstrates excellent performance in predicting the cell-type composition of spatial spots, through comprehensive benchmark and real-data experiments.
- SpaJoint demonstrates remarkable robustness to hyperparameters and significant advantage in computational efficiency.

## Supplementary Material

supplementary-bib-revison_bbag158

## Data Availability

Datasets analyzed in this paper are available in raw from their original authors (see Supplementary Tables 1 and 2). Meanwhile, backups of these datasets are also available at this link: https://drive.google.com/drive/folders/1RQKKQWv3ZkRbItMoOhQ3gwOwsbb5obd9?usp=drive_link. SpaJoint with a tutorial of installation and implementation, and the code on how to reproduce results in the manuscript are available at https://github.com/zichang96/SpaJoint and archived at the repository Zenodo (DOI: https://doi.org/10.5281/zenodo.15273724).

## References

[1] Ståhl PL, Salmén F, Vickovic S et al. Visualization and analysis of gene expression in tissue sections by spatial transcriptomics. *Science* 2016;353:78–82. 27365449 10.1126/science.aaf2403

[2] 10x Genomics , 2025. https://www.10xgenomics.com/platforms/visium (24 April 2025, date last accessed).

[3] Stickels RR, Murray E, Kumar P et al. Highly sensitive spatial transcriptomics at near-cellular resolution with Slide-seqV2. *Nat Biotechnol* 2021;39:313–9. 33288904 10.1038/s41587-020-0739-1 PMC8606189

[4] Eng C-HL, Lawson M, Zhu Q et al. Transcriptome-scale super-resolved imaging in tissues by RNA seqFISH+. *Nature* 2019;568:235–9. 30911168 10.1038/s41586-019-1049-y PMC6544023

[5] Chen KH, Boettiger AN, Moffitt JR et al. Spatially resolved, highly multiplexed RNA profiling in single cells. *Science* 2015;348:aaa6090. 25858977 10.1126/science.aaa6090 PMC4662681

[6] Wang X, Allen WE, Wright MA et al. Three-dimensional intact-tissue sequencing of single-cell transcriptional states. *Science* 2018;361:eaat5691. 29930089 10.1126/science.aat5691 PMC6339868

[7] Janesick A, Shelansky R, Gottscho AD et al. High resolution mapping of the tumor microenvironment using integrated single-cell, spatial and in situ analysis. *Nat Commun* 2023;14:8353. 38114474 10.1038/s41467-023-43458-x PMC10730913

[8] Stuart T, Butler A, Hoffman P et al. Comprehensive integration of single-cell data. *Cell* 2019;177:1888–902. 31178118 10.1016/j.cell.2019.05.031 PMC6687398

[9] Dong R, Yuan G-C. SpatialDWLS: accurate deconvolution of spatial transcriptomic data. *Genome Biol* 2021;22:145. 33971932 10.1186/s13059-021-02362-7 PMC8108367

[10] Elosua-Bayes M, Nieto P, Mereu E et al. SPOTlight: seeded NMF regression to deconvolute spatial transcriptomics spots with single-cell transcriptomes. *Nucleic Acids Res* 2021;49:e50. 33544846 10.1093/nar/gkab043 PMC8136778

[11] Ma Y, Zhou X. Spatially informed cell-type deconvolution for spatial transcriptomics. *Nat Biotechnol* 2022;40:1349–59. 35501392 10.1038/s41587-022-01273-7 PMC9464662

[12] Zhou Z, Zhong Y, Zhang Z et al. Spatial transcriptomics deconvolution at single-cell resolution using Redeconve. *Nat Commun* 2023;14:7930. 38040768 10.1038/s41467-023-43600-9 PMC10692090

[13] Moriel N, Senel E, Friedman N et al. NovoSpaRc: fexible spatial reconstruction of single-cell gene expression with optimal transport. *Nat Protoc* 2021;16:4177–200. 34349282 10.1038/s41596-021-00573-7

[14] Cable DM, Murray E, Zou LS et al. Robust decomposition of cell type mixtures in spatial transcriptomics. *Nat Biotechnol* 2022;40:517–26. 33603203 10.1038/s41587-021-00830-w PMC8606190

[15] Wan X, Xiao J, Tam SST et al. Integrating spatial and single-cell transcriptomics data using deep generative models with SpatialScope. *Nat Commun* 2023;14:7848. 10.1038/s41467-023-43629-w PMC10687049 38030617

[16] Kleshchevnikov V, Shmatko A, Dann E et al. Cell2location maps fine-grained cell types in spatial transcriptomics. Nat Biotechnol 2022;40:661–71. 35027729 10.1038/s41587-021-01139-4

[17] Lopez R, Li B, Keren-Shaul H et al. DestVI identifies continuums of cell types in spatial transcriptomics data. *Nat Biotechnol* 2022;40:1360–9. 35449415 10.1038/s41587-022-01272-8 PMC9756396

[18] Andersson A, Bergenstråhle J, Asp M et al. Single-cell and spatial transcriptomics enables probabilistic inference of cell type topography. *Commun Biol* 2020;3:565. 10.1038/s42003-020-01247-y PMC7547664 33037292

[19] Sun D, Liu Z, Li T et al. STRIDE: accurately decomposing and integrating spatial transcriptomics using single-cell RNA sequencing. *Nucleic Acids Res* 2022;50:e42. 35253896 10.1093/nar/gkac150 PMC9023289

[20] Rahimi A, Vale-Silva LA, Savitski MF et al. DOT: a flexible multi-objective optimization framework for transferring features across single-cell and spatial omics. *Nat Commun* 2024;15:4994. 10.1038/s41467-024-48868-z PMC11167014 38862466

[21] Biancalani T, Scalia G, Buffoni L et al. Deep learning and alignment of spatially resolved single-cell transcriptomes with tangram. *Nat Methods* 2021;18:1352–62. 34711971 10.1038/s41592-021-01264-7 PMC8566243

[22] Hao X, Wang S, Fang M et al. SPACEL: deep learning-based characterization of spatial transcriptome architectures. *Nat Commun* 2023;14:7603. 10.1038/s41467-023-43220-3 PMC10663563 37990022

[23] Song Q, Jing S. DSTG: deconvoluting spatial transcriptomics data through graph-based artificial intelligence. *Brief Bioinform* 2021;22:bbaa414. 33480403 10.1093/bib/bbaa414 PMC8425268

[24] Bae S, Na KJ, Koh J et al. CellDART: cell type inference by domain adaptation of single-cell and spatial transcriptomic data. *Nucleic Acids Res* 2022;50:e57. 35191503 10.1093/nar/gkac084 PMC9177989

[25] Wei R, He S, Bai S et al. Spatial charting of single-cell transcriptomes in tissues. *Nat Biotechnol* 2022;40:1190–9. 35314812 10.1038/s41587-022-01233-1 PMC9673606

[26] Vahid MR, Brown EL, Steen CB et al. High-resolution alignment of single-cell and spatial transcriptomes with CytoSPACE. *Nat Biotechnol* 2023;41:1543–8. 36879008 10.1038/s41587-023-01697-9 PMC10635828

[27] Li B, Tang Z, Budhkar A et al. SpaIM: single-cell spatial transcriptomics imputation via style transfer. *Nat Commun* 2025;16:7861. 10.1038/s41467-025-63185-9 PMC12375071 40849313

[28] Lin Y, Tung-Yu W, Wan S et al. scJoint integrates atlas-scale single-cell RNA-seq and ATAC-seq data with transfer learning. *Nat Biotechnol* 2022;40:703–10. 35058621 10.1038/s41587-021-01161-6 PMC9186323

[29] Miller BF, Huang F, Atta L et al. Reference-free cell type deconvolution of multi-cellular pixel-resolution spatially resolved transcriptomics data. *Nat Commun* 2022;13:2339. 10.1038/s41467-022-30033-z PMC9055051 35487922

[30] Long Y, Ang KS, Li M et al. Spatially informed clustering, integration, and deconvolution of spatial transcriptomics with GraphST. *Nat Commun* 2023;14:1155. 36859400 10.1038/s41467-023-36796-3 PMC9977836

[31] Li B, Zhang W, Guo C et al. Benchmarking spatial and single-cell transcriptomics integration methods for transcript distribution prediction and cell type deconvolution. *Nat Methods* 2022;19:662–70. 35577954 10.1038/s41592-022-01480-9

[32] Victora GD, Nussenzweig MC. Germinal centers. *Annu Rev Immunol* 2022;40:413–42. 35113731 10.1146/annurev-immunol-120419-022408

[33] Lam KC, Araya RE, Huang A et al. Microbiota triggers STING-type I IFN-dependent monocyte reprogramming of the tumor microenvironment. *Cell* 2021;184:5338–56. 34624222 10.1016/j.cell.2021.09.019 PMC8650838

[34] Bartoschek M, Oskolkov N, Bocci M et al. Spatially and functionally distinct subclasses of breast cancer-associated fibroblasts revealed by single cell RNA sequencing. *Nat Commun* 2018;9:5150. 30514914 10.1038/s41467-018-07582-3 PMC6279758

[35] Wu SZ, Al-Eryani G, Roden DL et al. A single-cell and spatially resolved atlas of human breast cancers. *Nat Genet* 2021;53:1334–47. 34493872 10.1038/s41588-021-00911-1 PMC9044823

[36] Hinton RB, Yutzey KE. Heart valve structure and function in development and disease. *Annu Rev Physiol* 2011;73:29–46. 20809794 10.1146/annurev-physiol-012110-142145 PMC4209403

[37] Shu S, Mengxia F, Chen X et al. Cellular landscapes of nondiseased human cardiac valves from end-stage heart failure—explanted heart. *Arterioscler Thromb Vasc Biol* 2022;42:1429–46. 36200446 10.1161/ATVBAHA.122.318314

[38] Litviňuková M, Talavera-López C, Maatz H et al. Cells of the adult human heart. *Nature* 2020;588:466–72. 32971526 10.1038/s41586-020-2797-4 PMC7681775

