# Supplementary material for: SpaJoint: a transfer learning method for spatial transcriptomics deconvolution
Source: Brief Bioinform. 2026 Apr 8;27(2):bbag158. doi: 10.1093/bib/bbag158 (PMC13069903; doi:10.1093/bib/bbag158)
Supplement: supplementary-bib-revison_bbag158 [file supplementary-bib-revison_bbag158.pdf]

Supplementary material for "SpaJoint: a transfer learning method for spatial transcriptomics deconvolution"

The supplementary material is organized as follows. Section S1 provides the computational details for evaluation metrics and methods. Section S2 provides comprehensive data descriptions (including simulated and real datasets). Section S3 presents extended experimental results, supplementing Section 3 in the manuscript. Section S4 contains downstream analysis on human breast cancer data.

S1 Benchmark metrics and methods

S1.1 benchmark metrics

For simulated datasets, we compare SpaJoint with nine integration methods. Each method generates a deconvolution matrix of $spots \times cell\ types$, as shown in Step3 of Fig. 1 in the manuscript. P_{tk} and $\hat{P}_{tk}$ represent the true and predicted proportion of cell type k in the t-th spot. We have performed normalization on the results to ensure that $\sum_{k=1}^{K} P_{tk} = 1$ and $\sum_{k=1}^{K} \hat{P}_{tk} = 1$, so a probability distribution of cell types can be acquired for each spot. We compared the deconvolution performance of different methods using four metrics: PCC, SSIM, RMSE, and JSD. Their definitions are as follows:

PCC (Pearson correlation coefficient) can measure the similarity between the estimated and true values, thus evaluating accuracy of the estimated deconvolution matrix. For one spot, a higher PCC value indicates better prediction accuracy. It is calculated by the following equation:

$$PCC = \frac{E[(\boldsymbol{P}_t - \boldsymbol{u}_t)(\hat{\boldsymbol{P}}_t - \hat{\boldsymbol{u}}_t)]}{\sigma_t \hat{\sigma}_t},$$

where $\boldsymbol{P}_t$ and $\hat{\boldsymbol{P}}_t$ are the true and predicted cell-type proportion vectors of the t-th spot, respectively; $\boldsymbol{u}_t$ and $\hat{\boldsymbol{u}}_t$ are the average proportion vectors of spot t in the ground truth and the predicted result, respectively; σ_t and $\hat{\sigma}_t$ are the standard deviations of the true and predicted cell type distribution in spot t, respectively.

SSIM (Structural Similarity) is a metric for image similarity, essentially comparing two matrices. Therefore, it can also be used to quantify the effectiveness of deconvolution. For one spot, a higher SSIM value indicates better prediction accuracy. The calculation is performed as follows:

$$SSIM = \frac{(2\boldsymbol{u}_t \hat{\boldsymbol{u}}_t + C_1^2)(2cov(\boldsymbol{P}_t, \hat{\boldsymbol{P}}_t))}{(\boldsymbol{u}_t^2 + \hat{\boldsymbol{u}}_t^2 + C_1^2)(\sigma_t^2 + \hat{\sigma}_t^2 + C_2^2)},$$

where $\boldsymbol{u}_t$, $\hat{\boldsymbol{u}}_t$, σ_t, $\hat{\sigma}_t$ are similar to those for calculating the PCC value; C_1 and C_2 are 0.01 and 0.03, respectively; $cov(\boldsymbol{P}_t, \widehat{\boldsymbol{P}}_t)$ is the covariance between the cell-type proportion vectors in the ground truth $\boldsymbol{P}_t$ and the predicted result $\widehat{\boldsymbol{P}}_t$.

RMSE (Root Mean Square Error) measures the dispersion between the true and predicted values. For one spot, a lower RMSE value indicates better prediction accuracy. The calculation is detailed below:

$$RMSE = \sqrt{\frac{1}{K}\sum_{k=1}^{K}(\boldsymbol{z}_{tk} - \hat{\boldsymbol{z}}_{tk})^2},$$

where $\boldsymbol{z}_{tk}$ and $\hat{\boldsymbol{z}}_{tk}$ are the z-scores of $\boldsymbol{P}_{tk}$ and $\widehat{\boldsymbol{P}}_{tk}$.

JSD (Jensen–Shannon Divergence) uses Kullback-Leibler divergence to measure the difference between two distributions. For one spot, a lower JSD value indicates better prediction accuracy. We calculate the JSD value for each spot using the following equations:

$$JSD = \frac{1}{2}KL\left(\boldsymbol{P}_t | \frac{\boldsymbol{P}_t + \widehat{\boldsymbol{P}}_t}{2}\right) + \frac{1}{2}KL(\widehat{\boldsymbol{P}}_t | \frac{\boldsymbol{P}_t + \widehat{\boldsymbol{P}}_t}{2}),$$

$$KL(\boldsymbol{a}|\boldsymbol{b}) = \sum_{k=1}^{K}(a_i + log\frac{a_i}{b_i}),$$

where $KL(\boldsymbol{a}|\boldsymbol{b})$ is the Kullback-Leibler divergence between two distributions $\boldsymbol{a}$ and $\boldsymbol{b}$.

To assess the relative accuracy of various integration methods, we can synthesize these four metrics. For a given dataset, we firstly computed the values of PCC, SSIM, RMSE and JSD across all spots predicted by each integration method. Subsequently, we ranked the PCC and SSIM values in descending order, assigning $RANK_{PCC}$ and $RANK_{SSIM}$ to each method. The method with the highest PCC or SSIM received the minimum rank (1), while the method with the lowest PCC or SSIM was assigned the maximum rank (N). Conversely, we ranked the RMSE and JSD values in ascending order, yielding $RANK_{RMSE}$ and $RANK_{JSD}$. Here, the method with the highest RMSE or JSD was given the maximum rank (N), and the method with the lowest RMSE or JSD received the minimum rank (1). Ultimately, we determined the **Mean Rank** for each integration method by taking the average as below:

$$Mean\ Rank = \frac{1}{4}(RANK_{PCC} + RANK_{SSIM} + RANK_{RMSE} + RANK_{JSD}).$$

For a dataset, the method with the first Mean Rank value has the best performance among all the integration methods.

All the above metrics were calculated at both the spot and cell-type levels. The above formulas are illustrated at the spot level, measuring the similarity between the

predicted and ground-truth vectors of cell-type proportion within each spot. When they are calculated at the cell-type levels, all calculations on the subscript t in metric formulas are changed to calculations on the subscript k, which quantify **the similarity between the predicted and true proportion vectors across spots for each cell type**. Taking PCC at the cell-type level as an example, the formula is:

$$PCC = \frac{E\left[(\boldsymbol{P}_k - \boldsymbol{u}_k)(\widehat{\boldsymbol{P}}_k - \widehat{\boldsymbol{u}}_k)\right]}{\sigma_k \hat{\sigma}_k},$$

where $\boldsymbol{P}_k$ and $\widehat{\boldsymbol{P}}_k$ are respectively the k-th column of the ground-truth matrix and deconvolution matrix, and the same notation applies to the remaining symbols. Note that, for brevity in the figure legends, we use "of location" and "of cluster" as shorthand for "at the spot level" and "at the cell-type level", respectively. In this way, we can compare the effectiveness of various deconvolution methods comprehensively.

S1.2 benchmark methods

To show the effectiveness of SpaJoint for ST cell type deconvolution with scRNA-seq data, we compared SpaJoint with nine state-of-the-art deconvolution methods, including RCTD, Spoint, Tangram, DOT, cell2location, CellDART, GraphST, CARD, and DestVI. we also included benchmarking comparisons with SpatialScope across all datasets and with STdeconvolve in three simulation datasets. Since SpatialScope is a method for cell type annotation of spots rather than deconvolution, while STdeconvolve is a reference-free method, we need to transform the outputs of these two methods so that they can be used for method comparison in this study.

SpatialScope consists of three steps. Firstly, it segments each spot into individual cells using the histology image. Secondly, it identifies the cell type for each spot, where the output can be used for comparison with our method. Finally, based on the first two steps, it decomposes spot-level data to obtain cell-level gene expression. As SpatialScope produced a cell type label for each spot rather than a deconvolution (spot × cell-type-presence-probability) matrix, we performed one-hot encoding on its output and converted it into a deconvolution matrix, to enable comparison with other deconvolution methods. Specifically, for each spot, we set the entry corresponding to its predicted cell type to 1 and all other cell-type entries to 0, yielding a binary 0-1 matrix.

STdeconvolve is a reference-free method, which means it does not require corresponding scRNA-seq data as input, and therefore cannot leverage cell-type-label information from single-cell data. It generates a spot × "no-ref cell type" (i.e., cell type 1 , cell type 2...) matrix, which can only identify the number of cell types but cannot assign specific label names, and a "no-ref cell type" × gene matrix, which can be used for subsequent annotation of real cell types. To make it comparable with the

deconvolution results in this study, we accurately annotated the results of STdeconvolve following the instructions in reference [1]. The specific steps were as follows: First, in the simulation, for the single-cell-resolution ST data with cell-type labels (before generating pseudo-spots), we generated the ground-truth transcriptional profiles of each cell type. Specifically, we averaged the gene expression of all spots belonging to the same cell type, then normalized at the cell-type level, and used this as the ground truth. Second, each no-ref cell type identified by STdeconvolve was matched with the ground-truth cell type that has the highest Pearson's correlation coefficient. This was done by computing the Pearson's correlation between every pair of no-ref-cell-type and ground-truth-cell-type transcriptional profiles. Real ST datasets lack ground-truth cell types, making it impossible to effectively annotate the output of STdeconvolve. This limitation is precisely why comparisons involving STdeconvolve can only be conducted on three simulated datasets with true cell-type labels.

Notably, to make the annotated deconvolution results of STdeconvolve comparable to other methods, we tuned its parameters so that the number of no-ref cell types equals the number of ground-truth cell types. Nevertheless, the final annotation may not cover all cell types, because several no-ref cell types may show the strongest correlation with the same ground-truth cell type; in such cases we assignd the same label to these no-ref cell types, causing the final annotated cell types fewer than the actual cell types. For example, in the mouse visual cortex STARmap dataset, the final annotation result obtained from STdeconvolve lacks two cell types "ExcitatoryL5" and "Micro".

S2 Dataset description

Mouse visual cortex STARmap data. For the simulation studies in Section 3.1 of the manuscript, we first constructed the simulated data based on a single-cell-resolution ST dataset acquired through STARmap sequencing as in the benchmarking study [2]. The cell type label of each spot in the dataset can be used as the ground truth when simulating a dataset with potentially ambiguous cell type assignations in each spot. The simulation method was to set a fixed window width, such as 750, to grid the spatial coordinates of the cells, and the cells divided into a window formed a pseudo-spot. The original dataset captured 1,549 cells, corresponding to 15 cell types from the mouse visual cortex. After gridding, the simulated data had 189 spots, with each spot containing 1-18 cells.

Mouse cortex seqFISH+ data. Then we employed the same scRNA-seq data as used in the mouse visual cortex study while replacing the matched ST data with seqFISH+ data. Given that seqFISH+ also provides single-cell-resolution ST data, we adopted a similar approach to that used for STARmap to establish the ground truth. Specifically, we

aggregated the original spots into 72 pseudo-spots using a window size of 500.

Mouse brain Stereo-seq data. Furthermore, to rigorously test the scalability and robustness of our method, we also incorporated a publicly available **large-scale** Stereo-seq dataset with high resolution, which was obtained from the postnatal day 7 (P7) murine whole brain sagittal section with 62968 spots and 23118 genes. We used the original Stereo-seq dataset as a spatially annotated single-cell reference and employed SRTsim [3] to generate synthetic ST data with ground truth. Using a sliding window size of 5, we generated 2,611 pseudo-spots, each encompassing 1-16 original spots.

Human lymph node 10x Visium data. The human lymph node presents a dynamic environment with various cell types spatially intermixed, posing a challenge for cell-type deconvolution. This dataset was acquired from human lymph node tissue containing germinal centers (GC). The scRNA-seq data used as reference was compiled from three studies on human secondary lymphoid organs, encompassing 34 cell types and 73,260 cells [4][5][6].

Mouse hippocampus Slide-seqV2 data. We used the hippocampus scRNA-seq dataset by Drop-seq [7] for deconvoluting the Slide-seqV2 dataset. The hippocampus primarily consists of three regions: the cornu ammonis 1 (CA1) region, the cornu ammonis 3 (CA3) region and the dentate gyrus.

Human breast cancer 10x Visium data. Breast cancer is a complex disease that arises from the uncontrolled growth of malignant cells in the breast tissue, with varying molecular and cellular characteristics among individual patients. We retrieved the scRNA-seq data as well as the ST data of primary pre-treatment breast tumor samples from a human breast cancer study [8].

Chicken heart development 10x Visium data. During early embryonic development, the heart initially forms as a simple tube and undergoes a series of intricate morphological changes, eventually developing into a fully functional four-chambered heart complete with the blood vessels. Research employed a combination of spatially resolved RNA sequencing and high-throughput single-cell RNA sequencing to investigate the development of the embryonic chicken heart [9][10].

Sources and more details for these seven datasets are provided in Supplementary Tables 1 and 2.

Supplementary Table 1: List of 7 spatial transcriptomics (ST) datasets and 5 single-cell RNA sequencing (scRNA-seq) datasets we used in our analysis. The scRNA-seq datasets were used as references to construct cell-type-specific profiles for ST datasets. The table contains dataset name (1st column), data type (2nd column), experimental platform (3rd column), year of publication (4th

column), number of genes (5th column), number of spots or cells (6th column), number of common HVGs (7th column), data source (obtained from the website link, see the bottom of the table, 8th column).

Dataset	Data Type	Protocol	Year	#Genes	#Spots /Cells	#Common HVGs	Link
Mouse visual cortex	Spatial	STARmap	2018	882	1549	170	*Link1
Mouse primary visual cortex (VISp)	scRNA	Smart-seq	2018	34041	14249	170	*Link2
Mouse cortex	Spatial	seqFISH+	2019	9784	574	283	*Link3
Mouse brain	Spatial	Stereo-seq	2022	23118	62968	791	*Link4
Human lymph nodes	Spatial	10X Visium	2019	36588	4035	486	*Link5
Human lymph nodes	scRNA	16S ribosomal RNA & 10X Chromium	2020	10237	73260	486	*Link6
Mouse hippocampus	Spatial	Slide-seq	2020	23265	17747	641	*Link7
Mouse hippocampus	scRNA	DropViZ	2018	27953	113507	641	*Link8
Human breast cancer	Spatial	10X Visium	2021	28402	4784	313	*Link9
Human breast cancer	scRNA	10X Chromium	2021	29733	6178	313	GSE176078
Chicken heart (D4)	Spatial	10X Visium	2021	24356	747	410	*Link10
Chicken heart (D4)	scRNA	10X Chromium	2020	24356	5653	410	GSE149457

***Link1:** https://drive.google.com/drive/folders/1pHmE9cg_tMcouV1LFJFtbyBJNp7oQo9J?usp=sharing
***Link2:** https://portal.brainmap.org/atlases-and-data/rnaseq/ mouse-v1-and-alm-smart-seq
***Link3:** https://github.com/CaiGroup/seqFISH-PLUS
***Link4:** https://db.cngb.org/stomics/datasets/STDS0000139/data
***Link5:** https://support.10xgenomics.com/spatial-gene-expression/datasets/1.0.0/V1 Human Lymph Node
***Link6:** https://cell2location.cog.sanger.ac.uk/paper/integrated lymphoid organ scrna/RegressionNBV4Torch 57covariates 73260cells 10237genes/sc.h5ad
***Link7:** https://singlecell.broadinstitute.org/single cell/study/SCP815
***Link8:** http://dropviz.org/
***Link9:** https://zenodo.org/record/4739739#.Ys0v6jdBy3D
***Link10:** https://github.com/madhavmantri/chicken_heart/tree/master/data

Supplementary Table 2: Chicken heart scRNA-seq and ST data information at D7, D10, D14. Other information is the same as D4 in Supplementary Table 1.

Dataset	Data Type	#Spots/Cells	#Common HVGs
Chicken heart (D7)	Spatial	1966	104
Chicken heart (D7)	scRNA	8463	104
Chicken heart (D10)	Spatial	1916	97
Chicken heart (D10)	scRNA	5190	97
Chicken heart (D14)	Spatial	1967	73
Chicken heart (D14)	scRNA	3009	73

S3 Additional experimental results

S3.1 performance comparison

Supp. Fig. 1-5 present additional comparative results across three simulated datasets and two real datasets.

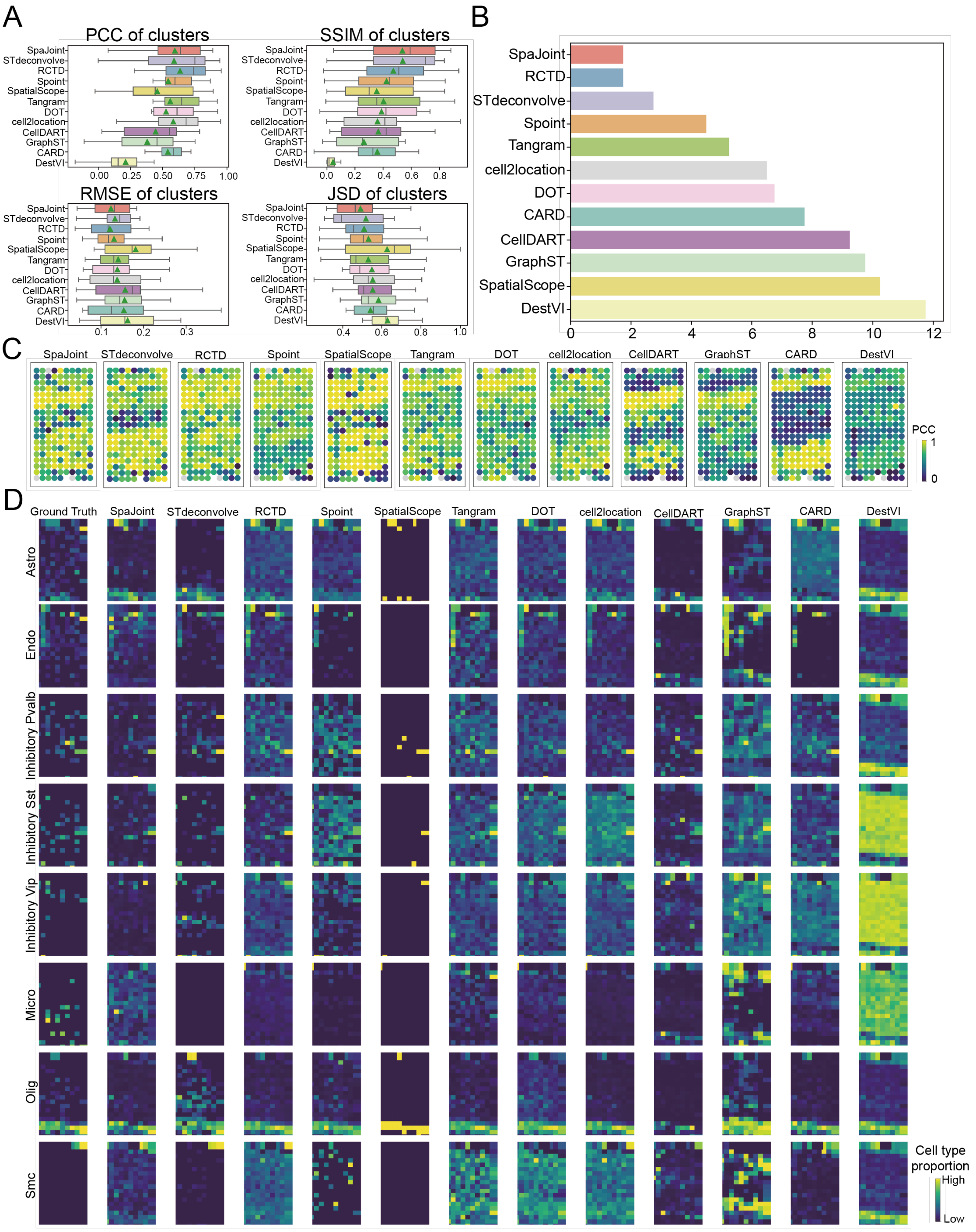

Supplementary Figure 1: Performance benchmarking with mouse visual cortex STARmap data. A. Boxplots of PCC, SSIM, RMSE and JSD of each deconvolution method, calculated at the cell-type levels. Center line: median; box limits: upper and lower quartiles; whisker: 1.5 × interquartile range; green triangle: mean value; number of cell types (clusters): 12. Higher PCC and SSIM, lower RMSE and JSD indicate better performance. **B.** Bar plots of Mean Rank (aggregated from PCC, SSIM, RMSE and JSD) at the cell-type level. **C.** PCC of spots from SpaJoint and 11 other methods. **D.** Proportions of the rest cell types in the spots, including the ground truth and the predicted results of 12 methods.

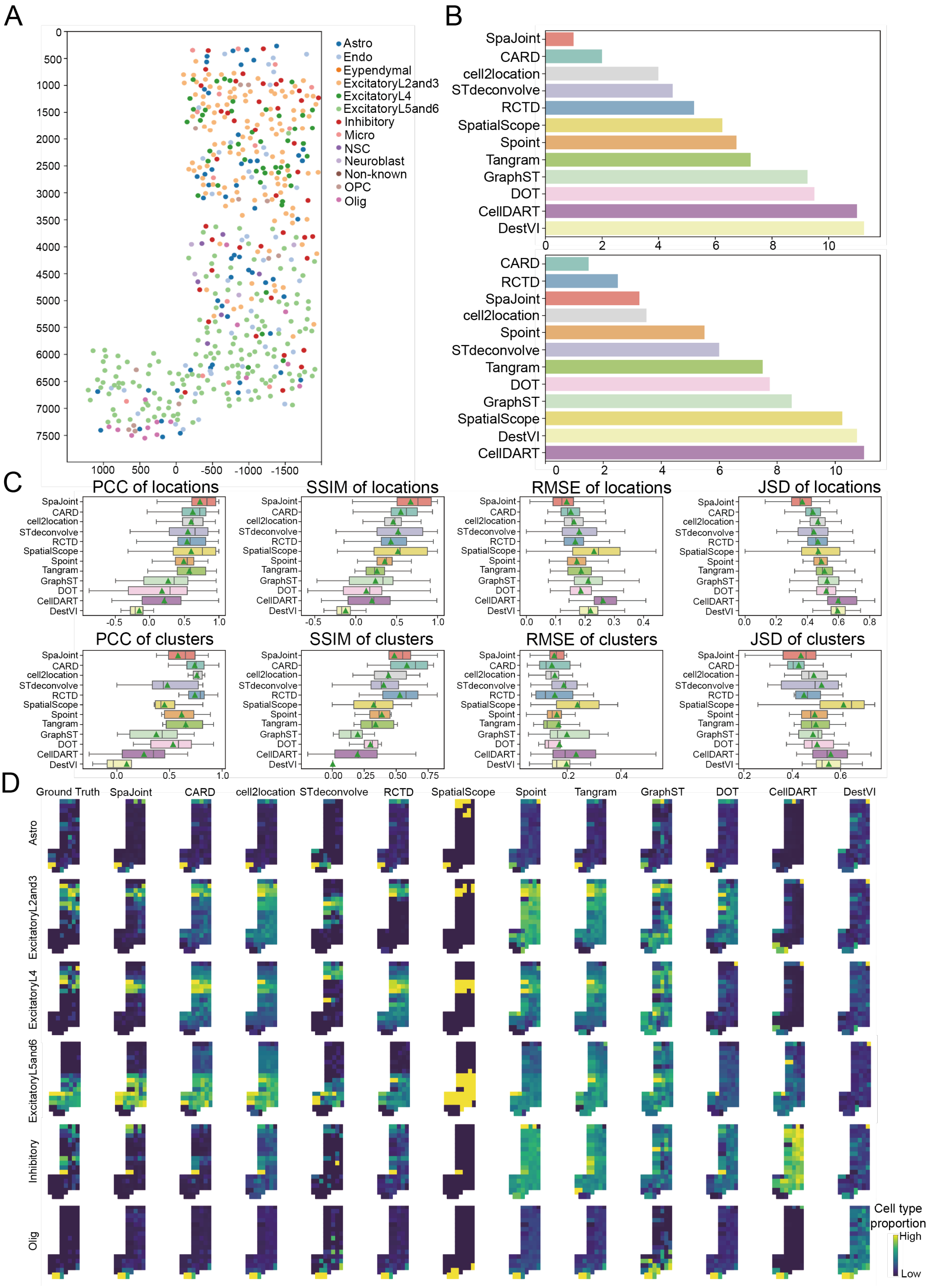

Supplementary Figure 2: Performance benchmarking with mouse cortex seqFISH+ data. A. The slide of mouse cortex with spots annotated by cell types. **B-C.** Bar plots of Mean Rank at the spot and cell-type level (aggregated from PCC, SSIM, RMSE and JSD). **D.** Boxplots of PCC, SSIM, RMSE and JSD of deconvolution methods in predicting the cell-type proportion of spots (locations) and spot distribution of cell types (clusters). Center line: median; box limits: upper and lower quartiles; whisker: 1.5 × interquartile range; green triangle: mean value; number of spots: 72; number of cell types: 8. **E.** The proportion of major cell types in the spots, including the ground truth and the predicted results of 12 methods.

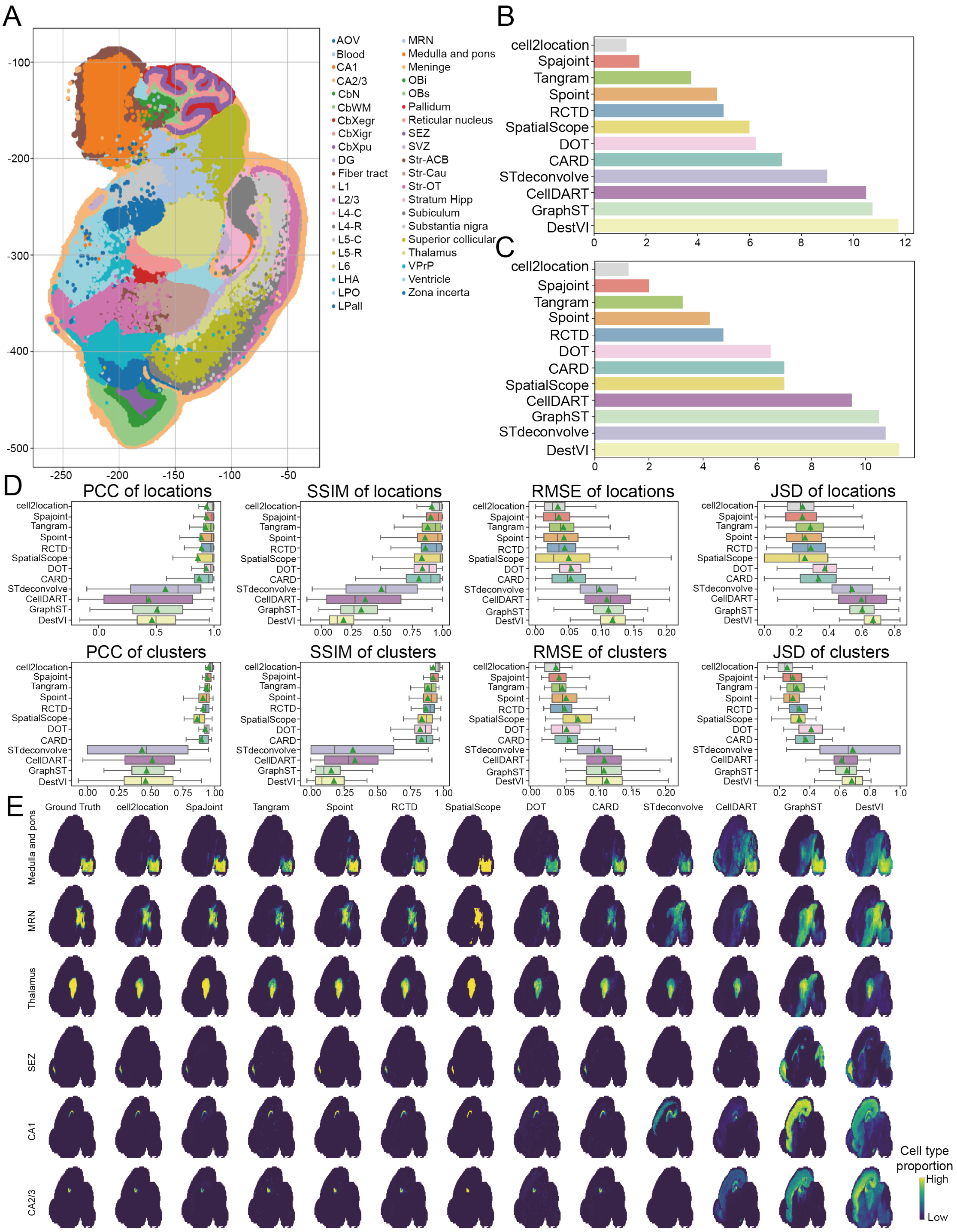

Supplementary Figure 3: Performance benchmarking with mouse brain Stereo-seq data. A. The slide of mouse brain with spots annotated by cell populations. **B-C.** Bar plots of Mean Rank at the spot and cell-type level (aggregated from PCC, SSIM, RMSE and JSD). **D.** Boxplots of PCC, SSIM, RMSE and JSD of each deconvolution method in predicting the cell-type proportion of spots (locations) and spot distribution of cell types (clusters). Center line: median; box limits: upper and lower quartiles; whisker: 1.5 × interquartile range; green triangle: mean value; number of spots: 2611; number of cell types: 41. **E.** The proportion in spots of the 3 most and 3 least abundant cell types in the original data, including the ground truth and the predicted results of 12 methods.

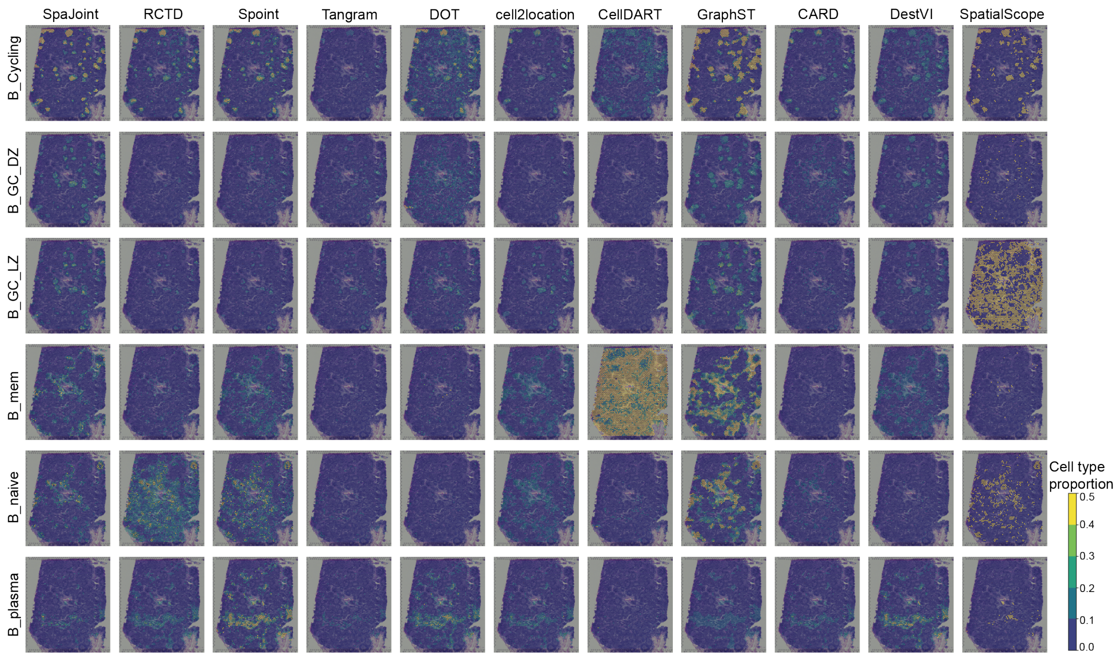

Supplementary Figure 4: Performance comparison on human lymph node data. The proportion in spots of subtypes of B cells, namely B_Cycling, B_GC_DZ, B_GC_LZ, B_mem, B_naive, and B_plasma, predicted by 11 methods.

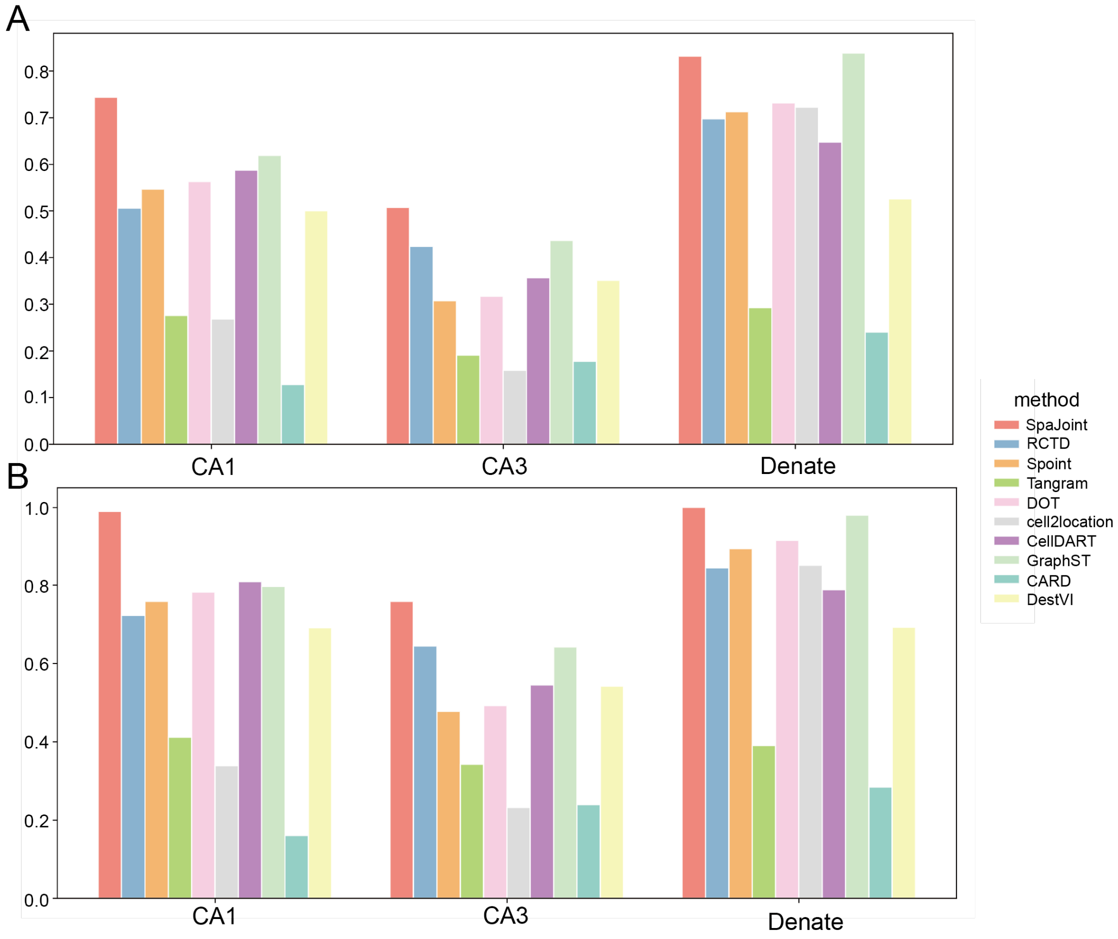

Supplementary Fig 5: Performance comparison on mouse hippocampus data. The mean predicted proportion of 3 major cell types on the spots where the expression of corresponding marker genes is **A.** greater than 0, and **B.** greater than 2.

Supp. Table 3 provides the max CPU and GPU memory usage of all methods on the human lymph node data.

Supplementary Table 3: The max CPU and GPU memory usage of SpaJoint and other 11 methods on the human lymph node data.

Method	Max CPU memory (MB)	Max GPU memory (MB)
SpaJoint	4909	4767
Spoint	10018	2659
Tangram	8772	11427
GraphST	11881	11333
CellDART	3238	839
cell2location	9303	3913
DestVI	6805	2105
SpatialScope	4526	0
RCTD	22631	0
CARD	27144	0
DOT	8913	0
STdeconvolve	9407	0

S3.2 sensitivity analysis and ablation study

Supp. Table 4 provides the specific weight combinations employed in Section 3.4 of the manuscript.

Supplementary Table 4: The weight combinations of loss functions used in the sensitivity analysis of Fig. 7A.

Weights Combination	w_1 for $L_{reduction}^{scRNA}(\theta)$	w_2 for $L_{reduction}^{ST}(\theta)$	w_3 for $L_{cos}(\theta)$	w_4 for $L_{loc}(\theta)$
1	0.1	0.1	0.1	0.7
2	0.2	0.2	0.2	0.4
3	0.2	0.2	0.4	0.2
4	0.3	0.3	0.2	0.2
5	0.4	0.4	0.1	0.1

To more comprehensively demonstrate SpaJoint's robustness to hyperparameters, we have included sensitivity analysis results for the other two simulated datasets in Supp. Fig. 6.

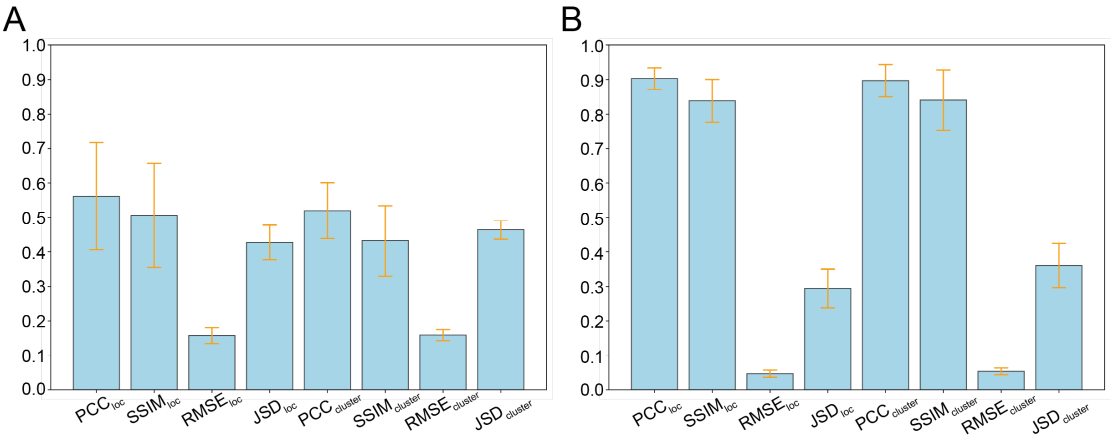

Supplementary Figure 6: Bar plots of eight different metrics (PCC, SSIM, RMSE, JSD for locations and clusters separately) for **A.** mouse cortex seqFISH+ data and **B.** mouse brain Stereo-seq data. For each hyperparameter combination, the mean of each metric over all locations or clusters is computed. The bar height is the average and the yelow error bar is the standard deviation across the means of 80 combinations.

To test how well SpaJoint can handle noisy or partially incorrect annotations, we conducted experiments to artificially introduce labeling errors in the mouse visual cortex STARmap data, by randomly shuffling a proportion (5% to 20%) of cell type labels in the reference scRNA-seq data. The result shown in Supp. Fig.7 demonstrates that SpaJoint remains desirably robust and tolerant to moderate annotation errors.

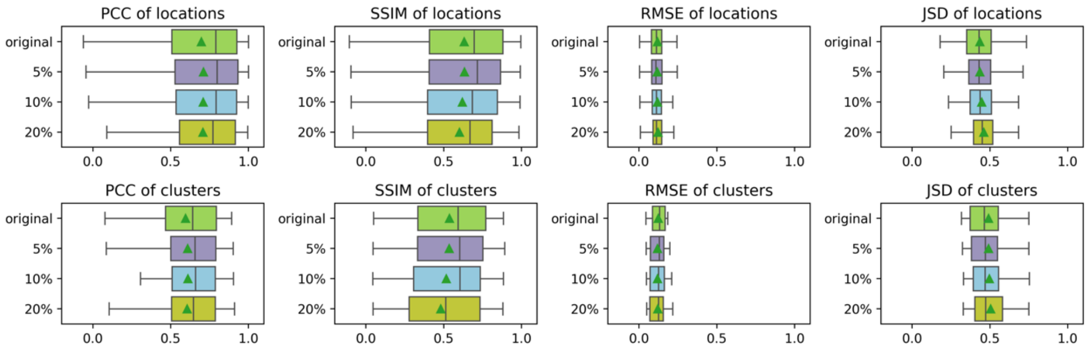

Supplementary Figure 7: Boxplots of PCC, SSIM, RMSE and JSD of each shuffle proportion (original means 0%) in predicting the cell-type proportion of spots, calculated at the location (spot) and cluster (cell-type) levels. Center line: median; box limits: upper and lower quartiles; whisker: 1.5 × interquartile range; green triangle: mean value; number of locations (spots): 189; number of clusters (cell types): 12. Higher PCC and SSIM, lower RMSE and JSD indicate better performance.

Apart from considering several numbers of HVGs (1000, 2000, 3000, 5000) in Fig. 7A, we also evaluated the outcomes using all genes. Regarding the number of HVGs specifically, we fixed the number of neighbors as 6 and the weights of loss functions as Weight 4 in Supp. Table 4, solely varying the number of HVGs. The results in Supp. Fig. 8 indicate that without gene selection, the overall accuracy of SpaJoint moderately decreases. This observation suggests that HVGs tend to enrich crucial biological signals,

whereas low-variable genes may contain "background noise", thus reducing the signal-to-noise ratio.

Furthermore, by systematically varying the number of selected HVGs, it is observed in Supp. Fig. 8 that the deconvolution accuracy shows only a marginal improvement with larger gene sets, despite a substantial increase in computational time. Therefore, selecting 2,000 HVGs was found to optimally balance accuracy and computational efficiency.

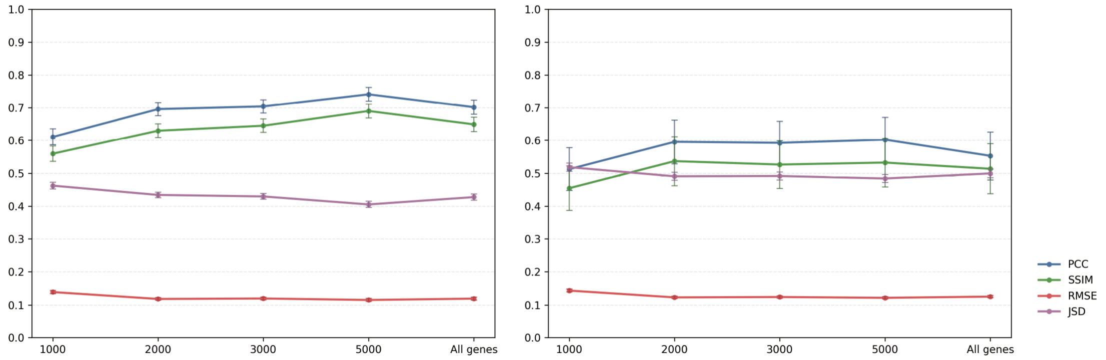

Supplementary Figure 8: Trend lines of four prediction metrics under different numbers of HVGs, with scatters on lines representing means and the error bars indicating standard errors. Higher PCC and SSIM, lower RMSE and JSD indicate better performance. Left: location level. Right: cell-type level.

To further investigate the impact of different loss components in the neural network, we particularly conducted a sensitivity analysis on weights of the loss functions (with weight combinations in Supp. Table 5, the number of HVGs fixed at 2,000 and the number of neighbors fixed at 6). The results shown in Supp. Fig. 9 indicate that the performance of SpaJoint remains notably stable with such weight variation.

Supplementary Table 5: The weight combinations of loss functions used in the additional sensitivity analysis.

Weights Combination	w_1 for $L_{reduction}^{scRNA}(\theta)$	w_2 for $L_{reduction}^{ST}(\theta)$	w_3 for $L_{cos}(\theta)$	w_4 for $L_{loc}(\theta)$
1	0.1	0.1	0.1	0.7
2	0.2	0.2	0.2	0.4
3	0.2	0.2	0.4	0.2
4	0.3	0.3	0.2	0.2
5	0.4	0.4	0.1	0.1
6	0.4	0.2	0.2	0.2
7	0.2	0.4	0.2	0.2
8	0.5	0.1	0.2	0.2
9	0.1	0.5	0.2	0.2

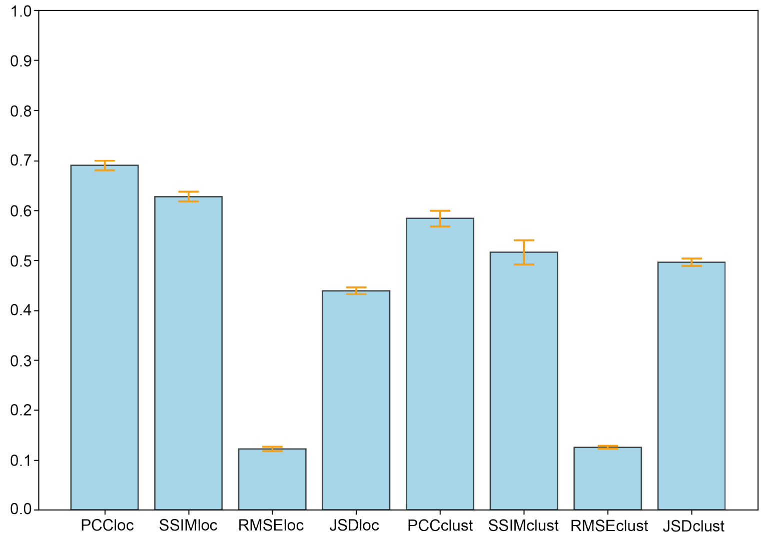

Supplementary Figure 9: Bar plots of eight different metrics (PCC, SSIM, RMSE, JSD for locations and clusters separately) with 9 weight combinations in Supp.Table 5. The bar height means the average and the error bar means the standard deviation.

Generally, the sparsity of scRNA gene expression matrices is very high, while the sparsity of ST gene expression matrices is relatively low. Therefore, we took the mouse visual cortex STARmap data as an example, adjusted the sparsity of its ST component, and conducted a series of ablation experiments; in this way we can examine how sparsity influences the result of ablation study.

The specific steps are as follows: First, the sparsity of scRNA and ST was evaluated by calculating the proportion of zeros in the gene expression matrix. It reveals that the sparsity of the scRNA gene expression matrix is 0.74, while that of ST is 0.3, which is the initial setup of our ablation study. Second, we gradually increased the sparsity of the ST gene expression matrix through binomial distributions. Explicitly, let the gene-expression count at each spot follow a binomial distribution, with the probability p $(0 < p \leq 1)$ maintaining the original value, and the probability $1 - p$ assigned value zero. Finally, we considerd four values of p: 1 (the initial setup in the manuscript), 0.8, 0.5, and 0.4, which correspond to the original ST sparsity of 0.3 and simulated ST sparsity of 0.44, 0.65, and 0.72, respectively. Notably, when p is set to 0.4, the sparsity of the ST part approximates that of the scRNA part.

We conductd a systematic ablation study on the scRNA and ST dimension reduction losses under four settings corresponding to $p = 1{,}0.8{,}0.5{,}0.4$; the results are shown in Supp.Fig. 10. Clearly, as the sparsity of the ST component increases, the impact of removing the scRNA reduction loss gradually grows, whereas the impact of removing the ST reduction loss diminishes. As the sparsity of the ST gene expression matrix approaches that of the scRNA matrix, the impact of separately removing scRNA and ST dimension reduction loss becomes increasingly similar (in Setting 4).

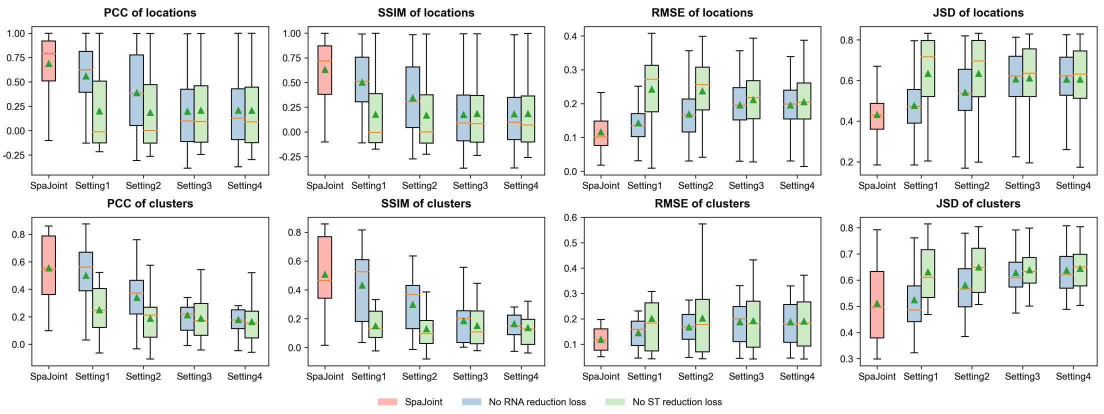

Supplementary Figure 10: The ablation study on the mouse visual cortex STARmap dataset comparing the original SpaJoint and four settings: four pairs of no scRNA reduction loss and no ST reduction loss in sequence with p = 1, 0.8, 0.5, 0.4. Boxplots of PCC, SSIM, RMSE, and JSD of the above 9 conditions in predicting the cell-type distribution of spots (locations) and spot distribution of cell types (clusters). Center line: median; box limits: upper and lower quartiles; whisker: 1.5 × interquartile range; green triangle: mean value. Higher PCC and SSIM, lower RMSE and JSD indicate better performance.

However, the sensitivity analysis results regarding weights in Supp. Table 5 indicate that although scRNA and ST reduction losses may differentially affect the outcomes due to their varying sparsity levels, such impacts exhibit minimal fluctuation in the final performance regarding accuracy (Supp. Fig. 9).

S4 Cell-cell communication analysis

In spatial transcriptomics studies, accurate deconvolution and cell-type annotation are the foundation of downstream analyses such as differential expression (DE) analysis and cell-cell interaction (CCI) inference.

For DE analysis, Fig. 4 in the manuscript has demonstrated that SpaJoint accurately delineates the mouse hippocampus. Based on Spajoint-derived cell-type annotations, the corresponding cell-type-specific marker genes are enriched expressed, confirming that Spajoint enables reliable identification of spatially resolved DE genes and thus improves DE analysis in ST data.

For CCI inference, extensive studies [11][12][13] have demonstrated that cell-cell communications are strongly correlated with the cells' spatial locations. Leveraging ST data to strengthen CCI analysis is therefore essential. To this end, we validated the performance of Spajoint in CCI analysis using human breast cancer data. Based on Spajoint's deconvolution result, we assigned each spot the cell type with the highest predicted proportion and generated a spatial plot of the cell-type annotation (Suppl. Fig. 11A). Ligand-receptor (L-R) pairs and the CCI analysis framework were obtained from CellPhoneDB v4.1.0 [14] (https://www.cellphonedb.org).

For both scRNA-seq and ST datasets, we performed CCI analysis between cell-type pairs, plotted the number of significantly expressed L-R pairs between cell types, in heatmaps and chord plots as shown in Supp. Fig. 11B, C. Compared with the results from scRNA-seq data, the ST data-based analysis reveals an increase in the total number of significant L-R pairs. Most remarkably, the scRNA-seq-based analysis only highlight interactions involving Epithelial cells, whereas the ST-SpaJoint-based analysis uncovers prominent Myeloid-associated interactions as well. Numerous breast cancer studies [15][16][17][18] have demonstrated that Myeloid cells actively participate in cellular communication during tumor progression, corroborating the accuracy of CCI analyses powered by ST data and Spajoint's annotation.

Moreover, published research [12] indicates that spatially co-localized cells are more likely to engage in interactions. The spatial plot (Supp.Fig. 11A) predicted by Spajoint reveals conspicuous co-localization of B cells with Myeloid cells, while both the heatmaps and chord plots (Supp. Fig. 11B, C) from SpaJoint show active L-R signaling between them. This observation further underscores the accuracy of Spajoint's annotation and its contribution to enhancing CCI performance.

Finally, focusing on L-R pairs that have been validated and functionally characterized in current breast cancer research, we plotted their mean expression and p-values across the cell-type pairs predicted by Spajoint in Supp. Fig. 11D (small populations of CAF and PVL were excluded). [15] demonstrated that interactions mediated by CSF1R as the receptor are active in Myeloid-targeted communications. [18] further confirmed that interactions in which ICAM1 serves as the ligand are active in Myeloid-sourced communications. These findings align perfectly with SpaJoint-based cell-type annotations and CCI results.

In summary, downstream analyses including cell-cell communication inference have further validated the excellent performance and biological interpretability of SpaJoint.

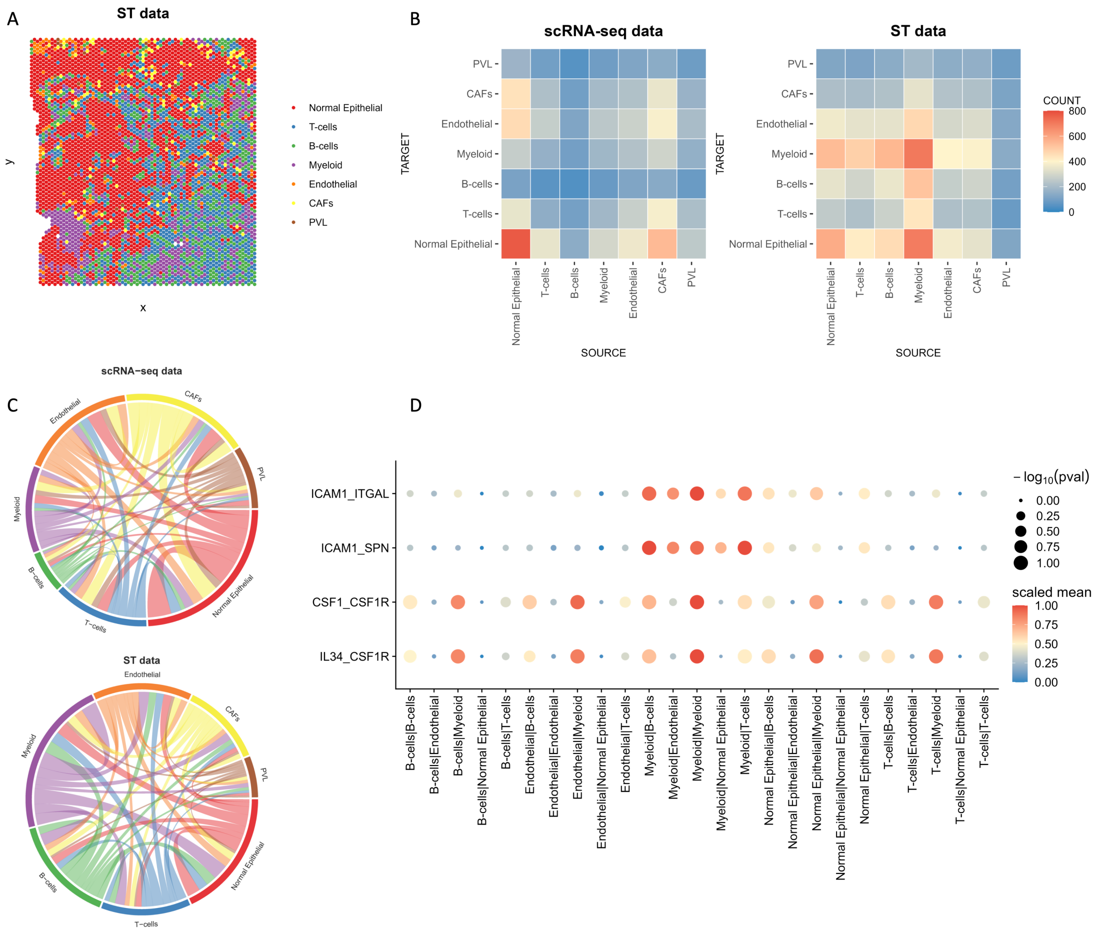

Supplementary Figure 11: Cell-cell communication analysis on human breast cancer data. A. Spatial dot plot of cell types annotated by SpaJoint. **B.** Color in heatmaps and **C.** Width of the ribbon in chord diagrams represents the number of significant L-R pairs between each cell-type pair derived from the scRNA-seq and ST data. **D.** Mean expression and significance levels of functionally characterized L-R pairs across major cell-type pairs. Color: the min-max-scaled mean expression; dot size: the min-max-scaled value of $-log_{10}(pvalue + 10^{-3})$.

References

[1] B. F. Miller, F. Huang, L. Atta, A. Sahoo, and J. Fan, “Reference-free cell type deconvolution of multi-cellular pixel-resolution spatially resolved transcriptomics data,” Nat. Commun., vol. 13, no. 1, p. 2339, Apr. 2022, doi: 10.1038/s41467-022-30033-z.

[2] Li, B., Zhang, W., Guo, C. *et al.* “Benchmarking spatial and single-cell transcriptomics integration methods for transcript distribution prediction and cell type deconvolution”. Natrue Methods, 19, 662–670, 2022. doi: 10.1038/s41592-022-01480-9.

[3] Zhu, J., Shang, L. & Zhou, X. SRTsim: spatial pattern preserving simulations for spatially resolved transcriptomics. Genome Biol., 24, 39, 2023. doi: 10.1186/s13059-023-02879-z.

[4] James KR, Gomes T, Elmentaite R, et al. Distinct microbial and immune niches of the human colon. Nat Immunol., 21(3):343-353, 2020. doi:10.1038/s41590-020-0602-z.

[5] Park JE, Botting RA, Domínguez Conde C, et al. A cell atlas of human thymic development defines T cell repertoire formation. Science. 367(6480): eaay3224. 2020. doi:10.1126/science.aay3224.
[6] King HW, Orban N, Riches JC, et al. Single-cell analysis of human B cell maturation predicts how antibody class switching shapes selection dynamics. Sci Immunol., 6(56): eabe6291, 2021. doi:10.1126/sciimmunol.abe6291.
[7] Saunders A, Macosko EZ, Wysoker A, et al. Molecular Diversity and Specializations among the Cells of the Adult Mouse Brain. Cell. 174(4): 1015-1030.e16. 2018. doi:10.1016/j.cell.2018.07.028.
[8] Wu SZ, Al-Eryani G, Roden DL, et al. A single-cell and spatially resolved atlas of human breast cancers. Nat Genet. 53(9): 1334-1347. 2021. doi:10.1038/s41588-021-00911-1.
[9] Mantri M, Scuderi GJ, Abedini-Nassab R, et al. Spatiotemporal single-cell RNA sequencing of developing chicken hearts identifies interplay between cellular differentiation and morphogenesis. Nat Commun. 12(1): 1771. 2021. doi:10.1038/s41467-021-21892-z.
[10] Lu Y, Chen QM, An L. SPADE: spatial deconvolution for domain specific cell-type estimation. Commun Biol. 7(1):469. 2024. doi:10.1038/s42003-024-06172-y.
[11] X. Wang, A. A. Almet, and Q. Nie, “The promising application of cell-cell interaction analysis in cancer from single-cell and spatial transcriptomics,” Semin. Cancer Biol., vol. 95, pp. 42–51, Oct. 2023, doi: 10.1016/j.semcancer.2023.07.001.
[12] R. Dries et al., “Giotto: a toolbox for integrative analysis and visualization of spatial expression data,” Genome Biol., vol. 22, no. 1, p. 78, Dec. 2021, doi: 10.1186/s13059-021-02286-2.
[13] S. Jin, M. V. Plikus, and Q. Nie, “CellChat for systematic analysis of cell-cell communication from single-cell and spatially resolved transcriptomics,” Nov. 05, 2023, Bioinformatics. doi: 10.1101/2023.11.05.565674.
[14] M. Efremova, M. Vento-Tormo, S. A. Teichmann, and R. Vento-Tormo, “CellPhoneDB: inferring cell–cell communication from combined expression of multi-subunit ligand–receptor complexes,” Nat. Protoc., vol. 15, no. 4, pp. 1484–1506, Apr. 2020, doi: 10.1038/s41596-020-0292-x.
[15] J. I. Griffiths et al., “Cellular interactions within the immune microenvironment underpins resistance to cell cycle inhibition in breast cancers,” Nat. Commun., vol. 16, no. 1, p. 2132, Mar. 2025, doi: 10.1038/s41467-025-56279-x.
[16] S. R. Dhruba et al., “Enhanced prediction of breast cancer patient response to chemotherapy by integrating deconvolved expression patterns of immune, stromal and tumor cells,” July 10, 2025, bioRxiv. doi: 10.1101/2024.06.14.598770.
[17] S. McAllister et al., “Cell Populations in Human Breast Cancers are Molecularly and Biologically Distinct with Age,” Oct. 15, 2024, In Review. doi: 10.21203/rs.3.rs-5167339/v1.
[18] E. Gonzalez et al., “Cancer systems immunology reveals myeloid—T cell interactions and B cell activation mediate response to checkpoint inhibition in metastatic breast cancer,” June 13, 2025, bioRxiv. doi: 10.1101/2025.06.09.658361.
